# Targeting of TAMs: can we be more clever than cancer cells?

**DOI:** 10.1038/s41423-024-01232-z

**Published:** 2024-11-08

**Authors:** Julia Kzhyshkowska, Jiaxin Shen, Irina Larionova

**Affiliations:** 1 Department of Innate Immunity and Tolerance, Institute of Transfusion Medicine and Immunology, Mannheim Institute for Innate Immunoscience (MI3), Medical Faculty Mannheim, University of Heidelberg, Theodor-Kutzer-Ufer, 1-3, 68167 Mannheim, Germany; 2 German Red Cross Blood Service Baden-Württemberg – Hessen, Friedrich-Ebert Str. 107, 68167 Mannheim, Germany; 3 Laboratory of Translational Cellular and Molecular Biomedicine, National Research Tomsk State University, 634050 Lenina av.36, Tomsk, Russia; 4 Bashkir State Medical University of the Ministry of Health of Russia, 450000 Teatralnaya Street, 2a, Ufa, Russia; 5 Department of Ultrasound in Medicine, The Second Affiliated Hospital of Zhejiang University School of Medicine, Zhejiang University, Hangzhou, 310009 China; 6 Laboratory of Molecular Therapy of Cancer, Cancer Research Institute, Tomsk National Research Medical Center, Russian Academy of Sciences, 634009 Kooperativnyi st, Tomsk, Russia

**Keywords:** Epigenetic, Metabolism, Scavenger receptor, Cytokine, Growth factor, Angiogenesis, Immunotherapy, Tumour immunology, Monocytes and macrophages

## Abstract

With increasing incidence and geography, cancer is one of the leading causes of death, reduced quality of life and disability worldwide. Principal progress in the development of new anticancer therapies, in improving the efficiency of immunotherapeutic tools, and in the personification of conventional therapies needs to consider cancer-specific and patient-specific programming of innate immunity. Intratumoral TAMs and their precursors, resident macrophages and monocytes, are principal regulators of tumor progression and therapy resistance. Our review summarizes the accumulated evidence for the subpopulations of TAMs and their increasing number of biomarkers, indicating their predictive value for the clinical parameters of carcinogenesis and therapy resistance, with a focus on solid cancers of non-infectious etiology. We present the state-of-the-art knowledge about the tumor-supporting functions of TAMs at all stages of tumor progression and highlight biomarkers, recently identified by single-cell and spatial analytical methods, that discriminate between tumor-promoting and tumor-inhibiting TAMs, where both subtypes express a combination of prototype M1 and M2 genes. Our review focuses on novel mechanisms involved in the crosstalk among epigenetic, signaling, transcriptional and metabolic pathways in TAMs. Particular attention has been given to the recently identified link between cancer cell metabolism and the epigenetic programming of TAMs by histone lactylation, which can be responsible for the unlimited protumoral programming of TAMs. Finally, we explain how TAMs interfere with currently used anticancer therapeutics and summarize the most advanced data from clinical trials, which we divide into four categories: inhibition of TAM survival and differentiation, inhibition of monocyte/TAM recruitment into tumors, functional reprogramming of TAMs, and genetic enhancement of macrophages.

## Introduction

Cancer,particularly its metastatic form, is one of the leading causes of death worldwide. According to World Health Organization data for 2019, cancer is the first or second leading cause of death in 112 countries under 70 years of age [1]. Cancer is an acute problem in both developed and developing countries due to the aging and growing populations, accelerated socioeconomic development and increased prevalence of associated risk factors (such as environmental pollution, chronic stress, and obesity). Worldwide, cancer is the leading cause of premature death and reduces life expectancy [2]. Currently, the treatment of solid tumors needs major improvement, despite significant progress in the development of immunotherapy approaches as well as the identification of a number of biomarkers, mostly genetic, that are useful for the personalization of conventional therapies [3–8]. Such principal improvement can be achieved if the potential of programming anticancer innate immunity, systemically or locally, is fully exploited.

Tumor-associated macrophages (TAMs) are key innate immune cells in the tumor microenvironment (TME) due to their quantity (in some tumors, TAMs constitute more than 50% of the tumor mass) and multiple tumor-supporting functions that act at all stages of carcinogenesis: tumor initiation, progression, metastatic spread via the blood or lymphatic circulation, response to therapy, and post-therapeutic metastatic relapse [9–11].

The major population of TAMs originates from circulating monocytes recruited by tumors, and recent studies have demonstrated that monocytes in cancer patients differ in their subpopulational content, transcriptome and metabolome from monocytes in healthy donors; moreover, subpopulational, transcriptomic and metabolomic signatures are highly specific for distinct solid tumors [12–16].

Substantial progress has been made in the accumulation of published evidence on the correlations between specific TAM subtypes and the clinical characteristics and therapeutic resistance of patients with solid cancers, and a deep understanding of the mechanism of TAM-mediated tumor support has led to a new wave of interest in the therapeutic targeting of TAMs.

Our review summarizes the knowledge about TAM subtypes and their biomarkers that correlate with primary tumor growth, metastatic spread and therapy resistance in several human solid cancers, with a focus on cancers of primarily noninfectious origin. We present the state-of-the-art in our understanding of TAM functions and secreted mediators that accelerate tumor progression, highlighting epigenetic, transcriptional and metabolic mechanisms in TAMs that offer new molecular interactions and pathways for reprogramming of TAMs. We also highlight the principal recent discoveries of novel biomarkers that discriminate between tumor-supporting and tumor-inhibiting TAMs independent of the M1/M2 dichotomy. Finally, our review summarizes major approaches for therapeutic targeting of TAMs that have been or are currently in clinical trials and evaluates their credibility and prospects for clinical application.

## Intratumor diversity of TAMs and their clinical significance in human cancers

**Fig. 1:**
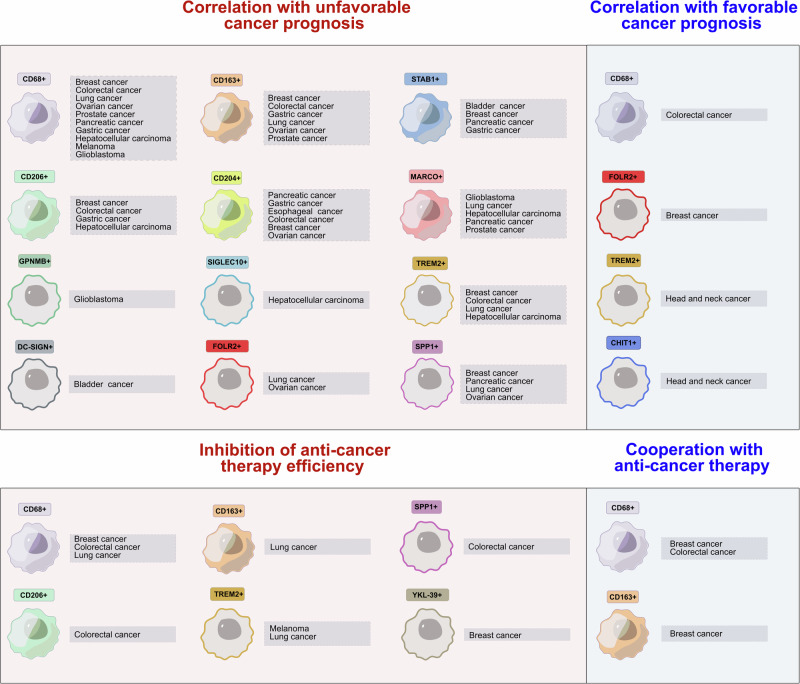
Clinical significance of TAMs. For the majority of TAM biomarkers, their correlation with unfavorable prognosis has been demonstrated for solid cancers. In contrast to other cancers, in colorectal cancer, CD68 (panmacrophage marker) is positively correlated with favorable patient prognosis. Individual TAM biomarkers are illustrated by different color coding. Filled macrophage icons are used as biomarkers for scavenger receptors. Сontoured macrophage icons are used as other TAM biomarkers. The orange background covers macrophage subtypes that correlate with a negative prognosis or with the inhibition of anticancer therapy efficacy. The blue background covers TAM subtypes that correlate with a favorable prognosis and TAMs that cooperate with anticancer therapy

The TAM phenotype reflects their specific role in different cancers. The correlations of distinct TAM subpopulations with primary tumor growth, lymphatic and hematogenous metastasis and treatment efficacy are specific for each type of cancer [9]. The major method originally applied for the identification of TAMs in human tumor tissue was immunohistochemistry (IHC) with anti-CD68 antibodies [17]. In the majority of studies, correlations of CD68 expression with the following clinicopathological parameters, histological grade, tumor size, TNM stage, lymph node status, and lymphovascular invasion, as well as with distant metastasis, recurrence and survival rates, were analyzed [9, 17]. Many studies have demonstrated that high infiltration of TAMs, as defined by CD68 expression, is associated with poor clinical outcomes in many cancers, including breast, lung, pancreatic, gastric, prostate, and ovarian cancers; hepatocellular carcinoma; melanoma; and glioblastoma [18] (Fig. 1). However, in contrast to other cancer types, the number of TAMs in colorectal cancer (CRC) is positively correlated with favorable outcomes, but the mechanism of this phenomenon remains unresolved. This observation was made in multiple international cohorts of patients with CRC: Swedish, Bulgarian, Japanese, Chinese, German, American, Greek, and others [19–24]. The number of CD68+ TAMs is decreased at advanced stages of CRC, in patients with regional LN metastases and distant metastases, and is associated with increased survival in CRC patients [19–24]. A recent study indicated that the association of high CD68+ TAM density with survival rate in patients with stage I-III CRC was dependent on tumoral T-cell density [25]. High TAM density was associated with a good prognosis in patients who also had high T-cell counts in tumors, while it was associated with an unfavorable prognosis in patients with low T-cell counts [25].

The development of quantitative histology equipped with a digital imaging system as well as multiple IF stains visualized by confocal microscopy has enabled more precise quantification of TAMs and identification of subpopulations and their localization in intratumoral compartments [21, 26, 27]. The most popular biomarkers of TAM subpopulations belong to the scavenger receptor family and include CD206, CD163, stabilin-1, MARCO, CD36, and CD204 [9, 28]. They are expressed in CD68+ macrophages in tumor tissue [15, 29–33]. CD206 and CD163 are the most frequently used markers of M2-skewed TAMs and are correlated with metastasis and poor disease outcomes in many cancer types, including CRC [9, 34–36]. CD204-expressing TAMs are found in gastric, colorectal, breast, lung, ovarian, pancreatic and esophageal cancers; MARCO+ TAMs correlate with poor prognosis, especially in lung, hepatocellular, breast and pancreatic cancers; and high stabilin-1 expression is associated with poor patient survival in patients with pancreatic, gastric and bladder cancers [9, 37]. Among the potentially predictive TAM markers PD-L1, YKL-39, YKL-40, TIE2, TREM-1, CCL18, Siglec1, and SPP1 have also been proposed [9, 38–41] (Fig. 1). More detailed information about clinically significant TAM biomarkers and their key protumor molecular functions is summarized in our recent reviews [9, 28, 37, 42].

The foremost challenge in anticancer treatment is multidrug resistance, resulting in the development of metastasis during therapy or during the follow-up period [43]. Major TAM subtypes are correlated with metastasis in the majority of solid cancers [44–46]. A high number of CD68+ TAMs is correlated with hematogenous metastasis and lymph node metastasis in breast, lung, prostate, ovarian, prostate, esophageal, bladder, and renal cancers [47–51]. However, increased numbers of CD68+ TAMs can negatively correlate with hematogenous and lymphatic metastasis, with most documented examples in colorectal cancer [19, 52, 53]. Increased amounts of specific TAM subsets identified by CD163, CD206, stabilin-1, and TREM2 biomarkers correlate with both hematogenous and lymphatic metastasis in all cancer types studied [21, 34, 47, 50, 54–58]. Even in CRC, the presence of M2 macrophages, defined by CD206, CD163, and stabilin-1 expression, is indicative of metastasis [35, 36, 59]. Extended information can be found in recent reviews [9, 45, 46].

TAMs can acquire different functional phenotypes depending on their localization in intratumoral compartments, for example, in hypoxic or perivascular regions, within tertiary lymphoid structures, in the tumor nest or at the invasive front [9, 37]. The histological location of TAMs can affect patient prognosis. Pancancer transcriptomic analysis revealed that higher expression of the TIM4+ TAM signature located in the tumor nest was associated with significantly worse disease-free survival (DFS) and overall survival (OS), whereas higher expression of the TIM4+ TAM signature in tertiary lymphoid structures predicted significantly better DFS [60]. High heterogeneity of TAMs has been identified in distinct morphological compartments in human breast cancer [61–63]. TAMs in the human breast cancer parenchyma are negatively correlated with lymphatic metastasis after neoadjuvant chemotherapy. Hypoxic macrophages are well described in glioblastoma [64–66]. Hypoxic niches may reprogram TAMs to an immunosuppressive state, whereas hypoxia-induced TAMs can destabilize endothelial adherent junctions, impairing drug delivery [64, 65]. In the last several years, single-cell RNA sequencing (scRNA-seq) and spatial transcriptomics (ST) technologies have revolutionized the field of TAM identification [37, 67, 68].

ST combined with scRNA-seq enables the identification of transcriptomes of individual cells in the context of tissue architecture [69]. ST methods can be useful in identifying specific cell‒cell interactions formed by targeting TAM subpopulations, which can be valuable for the development of new immunotherapeutic drugs. FDA-approved immunotherapy aims to reactivate suppressed immune components via checkpoints [70]. However, limitations in the efficiency of this class of drugs are challenging in regard to clinical applications [71]. The limitations in the clinical efficacy of currently used immunotherapy may be due to the immunosuppressive mechanisms executed by TAMs [72, 73]. There is a large expectation that TAM-targeted therapies can increase efficiency and personalize prescriptions to increase the spectrum of targeted immunotherapy tools. Using spatial transcriptomics analysis, several key recent findings regarding essential TAM–TME interactions were identified. For example, potential mechanisms of immune checkpoint inhibitor (ICI) resistance were demonstrated in two independent studies on colorectal cancer and hepatocellular carcinoma via 10x Genomics Visium ST technology [74, 75]. In these tumors, a histological barrier formed by the interaction of cancer-associated fibroblasts (CAFs) and SPP1+ macrophages decreases immunotherapy efficacy by limiting cytotoxic immune cell infiltration into malignant regions [74, 75]. In patients who respond to ICIs, SPP1+ macrophages/CAFs and immune cells interact easily [74, 75]. Another ST technology, NanoString GeoMx-DSP, was applied to examine the molecular mechanisms accompanying CRC development across normal mucosa, low-grade/high-grade dysplasia and cancer [76]. Dynamic changes are identified during the transition from normal tissue to dysplasia and from dysplasia to tumors [76]. There is increased infiltration of myeloid cells and a shift in macrophage populations from proinflammatory HLA-DR+ CD204- macrophages to HLA-DR-CD204+ immune-suppressive subsets [76]. Tumor samples from metastatic melanoma patients treated with ICIs were analyzed via GeoMx-DSP [77]. In the CD68+ compartment, PD-1 and HLA-DR expression in pretreated samples was significantly associated with resistance to therapy and poor survival, respectively [77]. We recently identified a new TAM-expressed marker for unfavorable prognosis in colon cancer—a regulator of glycolysis, PFKFB3 [14]. Using NanoString GeoMx, we found that in colon tumor tissue, PFKFB3 expression was linked to TAM accumulation and M2 polarization. PFKFB3 mRNA expression is negatively correlated with relapse and poor OS and PFS in colon cancer patients [14].

In our recent review, we thoroughly analyzed TAM phenotypic diversity in human cancers on the basis of the results of single-cell RNA-seq and ST technologies [37]. New functional biomarkers, including TREM2, MARCO, SPP1, C1QC, SIGLEC1, SIGLEC10, DC-SIGN, APOC1, CTSB, GPNMB, FOLR2 and others, annotated with immunosuppression, lipid metabolism, scavenging, antigen presentation, glycolysis, angiogenesis, hypoxia, and tumor cell invasion, have been defined for TAMs in different cancers [37]. Classical biomarkers of TAMs, including MRC1, CD163, MARCO, MAFB, and stabilin-1, comprise the gene signatures for novel TAM subsets. Several specific TAM subpopulations strongly correlate with disease outcome [37] (Fig. 1). According to recently collected data, TREM2, a surface lipid receptor, can be proposed as a new unprecedented macrophage biomarker with prominent immunosuppressive activity [78–80]. The number of TREM2+ TAMs is correlated with unfavorable survival or with a worse response rate to PD-1-based immune checkpoint inhibitors in patients with colorectal cancer, lung cancer, hepatocellular carcinoma, or melanoma [80–85]. The role of SPP1-expressing TAMs in tumor progression is under intensive investigation. Increased expression of SPP1 predicts poor prognosis in esophageal cancer, colorectal cancer, lung cancer, ovarian cancer, and glioma [41, 86–90]. GPNMB-expressing TAMs are specifically indicative of glioblastoma and are associated with poor prognosis in glioblastoma patients [91, 92].

Another state-of-the-art classification suggested for TAMs is based on multiomics data reflecting the molecular diversity of TAMs in mice and humans. The authors proposed a new consensus model of TAM subsets, including the following types of macrophages in cancer: interferon-primed TAMs (IFN-TAMs), immune regulatory TAMs (Reg-TAMs), inflammatory cytokine-enriched TAMs (Inflam-TAMs), lipid-associated TAMs (LA-TAMs), proangiogenic TAMs (Angio-TAMs), RTM-like TAMs (RTM-TAMs), and proliferating TAMs (Prolif-TAMs) [67]. In the most recent study, a comprehensive atlas of TAMs containing 23 clusters was identified by collecting scRNA-seq data from 17 human tumor types [93]. Some specific macrophage subsets are associated with the response to immune checkpoint inhibitors [93]. The expression profiles of TAM subpopulations dissected by scRNA-seq revealed tumor-specific phenotypes that cannot be classified according to the M1/M2 dichotomy; despite being convenient and commonly used, the understanding of the functions and mechanisms of action of TAMs is limited. The current task for translational oncology is the molecular targeting of particular functions of TAMs and the delivery of drugs to detrimental and decision-making TAM subsets [94]. For example, the targeting of TREM2, SPP1 and MARCO is currently under extensive investigation, and the first in vivo studies demonstrated promising results associated with the inhibition of immunosuppression and further decreased tumor growth [86, 95, 96]. However, in the case of MARCO, the specificity of targeting MARCO+ TAMs but not alveolar macrophages is a critical issue. MARCO is expressed on macrophages in healthy human lungs and is responsible for the uptake of dust and other pollutant particles and bacteria from the environment [97]. If this silent cleaning function is blocked, a localized inflammatory reaction in the lungs may develop on pollutant particles and bacteria, or the pollutant may enter the bloodstream and lead to systemic inflammation up to sepsis, and autoantibodies to MARCO lead to severe inflammatory lung disease [98].

TREM2 deficiency or anti-TREM2 targeting in combination with anti-PD-1 therapy diminishes tumor growth, promotes tumor regression, and induces a proinflammatory program in macrophages in vivo [85, 99]. In lung cancer, TREM2+ monocyte-derived TAMs reduce NK cell activity by modulating interleukin (IL)-18/IL-18BP decoy interactions and IL-15 production [100]. A novel therapeutic option was also based on the combination of anti-TREM2 antibodies and an NK cell-activating agent, resulting in the inhibition of tumor growth in a lung cancer model [100]. Blockade of SPP1 with an RNA aptamer strongly inhibited tumor growth and tumor infiltration by CD206+ and F4/80+ macrophages in xenograft mouse models [86]. However, there is a striking example that TREM2+ giant TAMs are correlated with good prognosis in head and neck squamous carcinoma patients [101] (Fig. 1). Multinucleated giant macrophages (MGC) are associated with a favorable prognosis in treatment-naive and preoperative chemotherapy-treated patients, and MGC density increases in tumors following preoperative therapy. Functionally, MGC seems to have an active program of foreign body response to the extracellular cluster of keratin, and MGC also expresses CHIT1, a chitotriosidase (or chitinase) with highly conserved hydrolytic activity [102]. The foreign body reaction of giant cells is associated with improved OS in patients with esophageal squamous cell carcinoma (ESCC) who receive preoperative chemoradiation therapy [103]. Despite the promising effects of TREM2 targeting in cancer, in obese cancer patients with metabolic disorders, TREM2+ TAM targeting can lead to adverse effects. TREM2 deficiency in this group of patients can lead to systemic hypercholesterolemia, body fat accumulation, and glucose intolerance [104]. It is related to the major function of TREM2, which is the regulation of tissue-level lipid homeostasis, suggesting that TREM2 is a key sensor of metabolic pathologies.

In the following chapters, the key tumor-supporting activities and molecular mechanisms of protumoral TAM programming are elucidated, and the results of most advanced clinical trials focused on TAM targeting are summarized and discussed.

## Tumor-supporting functions of TAMs

### TAM ontogeny and tumor initiation

**Fig. 2:**
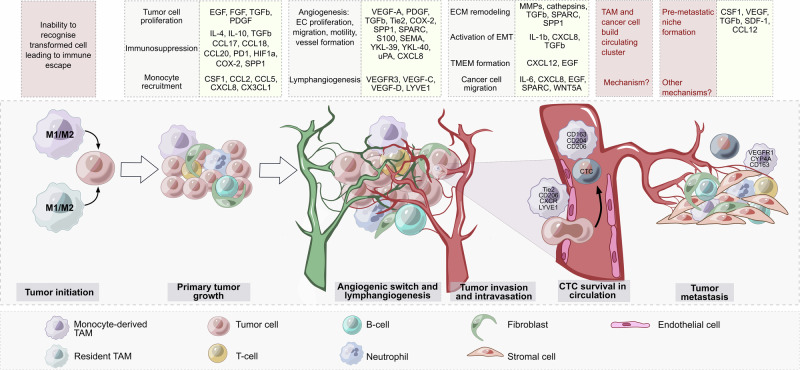
Functions and mediators of TAMs in tumor progression. Both resident macrophages and monocyte-derived macrophages can be involved in tumor initiation. Low-grade chronic inflammation drives genomic instability in cells. After the transformed cells escape immune control by macrophages, the growing cancer cell clones start recruiting blood monocytes. Other steps of tumor progression are controlled to a greater extent by monocyte-derived TAMs. TAMs secrete diverse protumor mediators (yellow boxes), which control many processes (gray boxes). TAMs are able to form an immunosuppressive microenvironment to facilitate angiogenesis and lymphangiogenesis, activate molecular mechanisms accompanying tumor invasion and intravasation, support cancer cell survival in the bloodstream, and help to form a premetastatic niche. Many open questions remain regarding the precise mechanisms involved in the generation of circulating clusters consisting of circulating tumor cells (CTCs) and TAMs as well as the role of TAMs in premetastatic niche formation

Local chronic inflammation, particularly low-grade inflammation, where macrophages constantly produce low levels of proinflammatory cytokines and reactive oxygen species (ROS), can drive tumor initiation by promoting genomic instability in malignant cells and, at the same time, by interfering with the ability of resident macrophages to distinguish between healthy somatic cells and transformed cells [105, 106] (Fig. 2). After transformed cells escape the first level of security control by resident macrophages, the proliferating cancer cell clones gain control over the resident macrophages located in close proximity to each other and start recruiting immune cells, including blood monocytes. Intensive heterogeneity in the site of chronic inflammation is due to the migration of diverse immune cells and somatic cells, and the activation of somatic cells does not allow macrophages to differ from malignant ones in terms of normal status. With the intensive growth of primary cancer cell clones, macrophages start to recognize tumors as healthy tissue and will now follow the instructions of cancer cells. In this coevolving cancer system, TAMs help tumors grow and escape other levels of immune control [11] (Fig. 2).

Some pathogenic microorganisms and viruses are able to initiate tumor growth. For example, human papillomavirus (HPV) infection of mucosal tissue may lead to head and neck, cervical, penile, anal and vaginal cancers [107]; hepatitis B virus (HBV) infection may induce hepatocellular carcinoma development [108]; Epstein‒Barr virus (EBV) drives nasopharyngeal carcinoma [109]; Kaposi’s sarcoma-associated herpesvirus infection is associated with Kaposi’s sarcoma [110].

The majority of viruses employ monocytes/macrophages as repositories and cells for productive replication [111]. The underlying mechanism of TAM involvement in virus-induced tumor growth has not been fully established, and TAM associations with clinical outcomes in patients with virus-associated cancers have still not been fully investigated. However, we performed several experimental studies indicating the crucial role of TAMs in virus-associated tumors. Increased infiltration by macrophages into the epithelium of the cervix was observed along with the progression of cervical intraepithelial neoplasia to invasive cancer [112]. Compared with lesions that regressed, low-grade intraepithelial lesions that persisted or progressed presented increased numbers of macrophages [112]. In an HPV16 E6- and E7-expressing TC-1 mouse tumor model, depletion of TAMs inhibited tumor growth and stimulated the infiltration of tumors by CD8+ lymphocytes, indicating that TAMs can suppress the antitumor immune response in HPV+ tumors [113]. Excessive M2-polarized macrophage accumulation was found in virus-associated hepatocellular carcinoma (HCC) compared with nonviral HCC [114]. The authors suggested that hepatitis B virus (HBV) infection induces M2-TAM polarization, leading to the development of liver fibrosis and, subsequently, to the development of HCC [114]. Kaposi’s sarcoma herpes virus (KSHV) infection of endothelial cells in vitro elevated Ang-2 expression to promote the migration and recruitment of monocytes into virus-induced tumors as well as IL-6, IL-10, and IL-13 expression to facilitate the differentiation and polarization of monocytes into TAMs [115]. KSHV-induced TAMs enhanced tumor growth and promoted tumor angiogenesis in a mouse model [115]. *Helicobacter pylori* (*H. pylori*) infection triggers chronic inflammation that can be associated with gastric cancer [116]. Tumor-derived exosomes from infected gastric cancer cells containing mesenchymal‒epithelial transition factors are internalized into macrophages and educate the macrophages toward a protumorigenic phenotype [116].

TAMs are highly plastic, and their activities can strongly depend on the signals produced by cancer cells, the intratumor localization of TAMs, and their interactions with the cellular and structural components of the TME [11]. All these parameters interact in the context of specific cancer types and can be affected by both local and systemic metabolic and hormonal factors [10, 105]. Finally, the application of anticancer treatment significantly impacts the functions of TAMs [94, 117–119]. In addition to cancer-specific secreted factors, the mechanisms responsible for molecular TAM heterogeneity are under intensive investigation [94]. In breast cancer, the adaptation of monocytes and macrophages to specific intertumoral locations within tumor regions can be a driver of TAM plasticity and heterogeneity [9, 120].

In addition to education by the TME, the ontogeny of macrophages also contributes to their heterogeneity [121]. The new paradigm of macrophage ontogeny confirmed that both embryonic tissue-resident macrophages originating from the yolk sac and fetal liver, as well as bone marrow monocyte-derived macrophages, constitute the TAM pool in tumor tissues [121–123]. Accumulated data demonstrate distinct transcriptional and functional programming of TAMs of different origins in breast, lung, ovarian, pancreatic, colorectal and brain cancers (reviewed by others) [121, 122]. In a mouse model of ovarian cancer carcinomatosis, CD163+Tim4+ omental macrophages were found to be of embryonic origin but not derived from bone marrow–dependent monocyte precursors [124]. The depletion of omental resident CD163+Tim4+ macrophages demonstrated that these cells play essential roles in tumor progression and the metastatic spread of disease in ovarian cancer [124]. In triple-negative breast cancer, tissue-resident macrophages are crucial cells that initiate tumor growth and facilitate recurrence and metastasis development [125]. In vivo, local depletion of mammary gland tissue-resident macrophages (MGTRMs) in mammary gland fat pads the day before cancer cell transplantation significantly reduces tumor growth and infiltration by TAMs, and depletion of MGTRMs at the site of tumor resection noticeably reduces recurrence and distant metastases and improves chemotherapy outcomes [125]. In contrast, FOLR2+ TAMs of resident origin in human breast cancer are associated with favorable prognosis [126]. FOLR2+ macrophages are located in the stroma in perivascular niches and interact with tumor-infiltrating CD8+ T cells, which is positively correlated with CD8+ T-cell activation and patient survival [126]. The folate receptor beta (FOLR2) is a TAM biomarker that enables discrimination between TAMs that can originate from resident macrophages and TAMs that originate from newly infiltrating monocytes [126]. The ability of FOLR2+ macrophages (which are also found in healthy mammary glands) to activate cytotoxic T cells in breast tumors is highly important and allows us to hypothesize that, at least in breast cancer, it is easier for cancer cells to program incoming monocytes to differentiate into tumor-supporting TAMs to reprogram resident tissue macrophages, which still retain the ability to fight against cancer. Pronounced tumor-supporting activity of TAMs was also found in a murine model of pancreatic ductal adenocarcinoma (PDAC), where protumoral IL-1beta+ TAMs were found to originate from monocytes but not from FOLR2+ resident macrophages [127]. Additionally, in human nonsmall lung cancer (NSCLC), the TAMs with the most pronounced tumor-supporting program have a monocyte origin [128]. However, tissue-resident macrophages support the formation of a protumorigenic niche in the early stages of NSCLC [129]. Another study discriminated between the distinct protumoral activities of TAMs of monocyte and resident macrophage origin, where monocyte-derived TAMs had increased antigen-presenting activity, whereas TAMs derived from resident macrophages of embryonic origin supported remodeling of the extracellular matrix [130]. Resident TAMs can have relatively stable programming that can be fixed at the epigenetic level; however, a certain level of reversibility is provided by the epigenetic enzymatic machinery. Using single-cell analysis of breast cancer tissue from patients and a PyMT breast cancer murine model, Ramos et al. identified novel biomarkers (and their combinations) to distinguish between pro- and antitumoral TAMs. CAMD1+ TAMs can be classified as protumoral on the basis of mouse studies, but their clinical correlations with the progression of human cancer remain to be identified [126]. However, a comparison of FOLR2+CAMD1- and FOLR2^low^CAMD1+ TAMs clearly revealed that both subpopulations of TAMs express a specific mixture of the porotype “M1” and “M2” genes, highlighting the limitations of the frequently used M1 and M2 terminology [126]. Identifying the full spectrum of antitumoral effects of FOLR2+ TAMs and determining whether FOLR2+ can mark macrophages with similar or disease-specific programming at the epigenetic, transcriptional and metabolic levels in other cancers and in other pathologies are highly important, and FOLR2 has already been suggested as a target for antibody-based cancer immunotherapy for acute myeloid leukemia [131]. Thus, in human lung adenocarcinoma, single-cell transcriptome profiling suggested that FOLR2+ TAMs, as has been shown for breast cancer, also originate from tissue-resident (in this case, alveolar) macrophages. However, FOLR2+ TAMs are enriched in more malignant invasive lung adenocarcinomas and are likely involved in CD4+ T-cell recruitment [132]. In the kidney, FOLR2+ macrophages can interact with fibroblasts to promote fibrosis [133]. FOLR2+ macrophages appear very early in development, and FOLR2+ macrophages in the yolk sac have antimicrobial protective effects via the activation of neutrophils [134]. In mice with experimentally induced endometriosis, FOLR2+ macrophages exhibit proangiogenic and profibrotic activity [135], which, in the case of cancer, support tumor growth. The similarity or heterogeneity between FOLR2+ macrophages in various pathologies is an open question, which single-cell analysis allows us to address at the level of molecular profiling. However, the functionality of FOLR2+ macrophages is only emerging, and the role of folate receptor beta itself in macrophages in distinct pathophysiological settings needs clarification.

Another example of a recently identified biomarker exclusively expressed on distinct subpopulations of TAMs is CXCL9 and SSP1 in head and neck squamous cell carcinoma (HNSCC) [136]. The ratio of CXCL9/SSP1 TAMs, defined by the authors as CS TAM polarity, is positively associated with the infiltration of T cells, B cells and DCs in HNSCC patients [136]. As in the case of FOLR2+ TAMs and CAMD1+ TAMs, the transcriptomes of CXCL9+ TAMs and SSP1+ TAMs did not correspond to the traditional M1 and M2 dichotomy, and both CXCL9 and SSP1 expressed mixtures of genes traditionally categorized as M0-, M1- and M2-specific biomarkers. CS TAM polarity is also found in patients with lung and colorectal cancer; however, whether CS polarity is linked to TAM origination from resident macrophages remains to be clarified. It is of interest to determine whether FOLR2+ cells specifically fall into the CXCL9 and SSP1 categories.

TAMs of a resident origin can be more proinflammatory and profibrotic and can support epithelial-mesenchymal transition (EMT). Monocyte-derived TAMs are more immunosuppressive and can be responsible for antigen presentation, extracellular matrix degradation, and tumor spreading [121]. Programming of monocyte-derived TAMs in solid tumors can start systemically at least one differentiation step before macrophage maturation—at the level of circulating monocytes [13–15, 137–140]. Circulating monocytes, depending on the type of cancer, change their phenotype, transcription and metabolic programs [14, 15, 139]. Our most recent study applied targeted mass spectrometry and demonstrated cancer-specific changes in amino acid profiles in monocytes of patients with primary cancer before therapy onset [139]. The most pronounced differences in amino acid metabolism between monocytes from cancer patients and monocytes from healthy donors were found in breast cancer patients (decreases in tryptophan and aspartic acid) and in ovarian cancer patients (decreases in citrulline). Such changes can be indicative of the immunosuppressive programming of monocytes already in circulation; however, it is unclear whether these changes are induced by cancer-derived factors or whether these changes occurred earlier and predisposed individuals to cancer development. Large cohort studies are needed to identify which factors (genetic, environmental, and lifestyle) can affect amino acid metabolism in monocytes, and such knowledge can be applied to identify targets in monocytes that block their ability to differentiate into protumoral TAMs. Considering that TAMs in CRC can retain the ability to restrict tumor progression, a significant increase in aspartic acid and citrulline was identified in the monocytes of patients with CRC compared with those of patients with other types of cancer [139].

Thus, not only tumor microenvironmental factors but also TAM ontogeny and TAM localization may play a role in the prevalence of TAM functionality. In the TME, TAMs are able to support primary tumor growth, angiogenesis, lymphangiogenesis, tumor cell invasion, survival of cancer cells in circulation, metastatic niche formation, and resistance to therapy [141–143] (Fig. 2).

### TAMs induce immunosuppression

In the TME, the major protumor function of TAMs is the production of diverse cytokines and growth factors that support the survival and proliferation of cancer cells, suppress their apoptosis, and increase their migratory potential needed for cancer cell invasion and extravasation into the circulation [144]. In established tumors, cancer cells reeducate TAMs to an immunosuppressive anti-inflammatory phenotype that supports tumor growth and facilitates tumor progression via the production of diverse tumor growth factors (e.g., EGF, FGF, TGFb, and PDGF), proangiogenic molecules (e.g., VEGF-A, SPP1, YKL-40, TIE2, and CXCL8), immunosuppressive factors (e.g., IL-10, PD-L1, CCL17, PGE, CCL20, and ROS), and matrix remodeling factors (e.g., matrix metalloproteinases [MMPs], uPAs, SPARC, and cathepsins) [17, 144, 145].

The second equally crucial activity of TAMs that is needed from the beginning of tumor development is the suppression of anticancer immune reactions (Fig. 2). TAMs can secrete large amounts of the immunosuppressive cytokine IL-10, which prevents the tumor cell-killing activity of CD8+ T cells, Th1 cells, and NK cells and activates Treg recruitment [142, 144, 145]. A number of immunosuppressive TAM subpopulations were revealed via single-cell analysis [37]. These TAM subsets include TREM2+ , MARCO+ , SPP1+ , CCL18+ , SIGLEC10+ , APOC1+ , IL10+ , and DC-SIGN+ macrophages, which are found in diverse types of cancer [37]. TAMs can suppress CD8+ cytotoxic T cells via the production of anti-inflammatory cytokines, inhibition of T-cell proliferation, or activation of T-cell checkpoint blockade through the engagement of inhibitory receptors. TAMs also express ligands for the inhibitory receptors programmed cell death protein 1 (PD-1) and cytotoxic T-lymphocyte antigen 4 (CTLA-4), which inhibit T-cell effector functions [145]. Immunosuppressive factors produced by TAMs in the TME include chemokines (e.g., CCL2, CCL5, CCL17, CCL18, CCL20 and CCL22), cytokines (e.g., HGF, PDGF-B, VEGF, IL-4, IL-10, and TGF-β) and enzymes (e.g., cathepsin K, COX-2, ARG1, and MMPs) [146]. In metastatic gastric cancer, the interaction of SPP1+ TAMs with CD8+ exhausted T cells via GDF15-TGFBR2 was demonstrated [147]. In the liver metastasis model, GDF15+  SPP1+ TAMs accumulated extensively in the metastatic site, but their amount was reduced after the blockade of GDF15 (a member of the TGFbeta superfamily). Inhibiting GDF15 significantly increased the infiltration of liver metastases by CD8+  T cells and reversed the immunosuppressive effect [147]. In a spontaneous BrafV600E-driven mouse melanoma model, specific depletion of CD163+ macrophages resulted in massive infiltration of activated T cells and significantly suppressed tumor growth [148]. After CD163+ TAM depletion, the remaining TAMs are re-educated toward the tumor-suppressing phenotype and express CD11c as well as the immune-modulatory molecules CIITA, CXCL9, and CD209D [148]. CD163+ exosomes derived from TAMs contribute significantly to immune suppression [149]. PD-1 is expressed by myeloid cells, including TAMs, in humans and mice [150]. TAM-derived exosomes activated by Rab27a carry high levels of PD-L1 and interact with stimulated, but not with unstimulated, CD8+ T cells, suppressing their proliferation and cytotoxic function in tumors. In a murine melanoma model, targeting macrophage RAB27A led to antitumor immune modulation and sensitized tumors to anti-PD-1 treatment [149]. The upregulation of Notch signaling in TAMs stimulates their immunosuppressive activity [151]. The combination of NOTCH1 signaling inhibition with anti-PD-1 therapy decreased tumor growth and activated the antitumor immune response in a mouse model of pancreatic cancer [151].

Myeloid-specific PD-1 targeting can play a decisive role in systemic antitumor responses [152]. In tumor-bearing mice, myeloid-specific rather than T-cell-specific PD-1 ablation more effectively decreased tumor growth and induced an increase in T-effector memory cells [152]. PD-1 expression on TAMs decreases phagocytic potency against tumor cells, whereas PD-1–PD-L1 blockade significantly increases phagocytosis by PD-1+ macrophages and reduces tumor growth in vivo [150]. Compared with T-cell-specific SHP-2 deletion, ablation of SHP-2, a regulator of PD-1 activity, in myeloid cells induced a decrease in tumor growth [153]. In tumor models of melanoma and fibrosarcoma, myeloid-specific SHP-2 ablation led to increased tumor infiltration by proinflammatory monocytes and concomitant recruitment and activation of CD4+ and CD8+ TEF cells. TAMs isolated from mice with myeloid-specific SHP-2 deletion presented increased expression of MHC II, CD86 and IFN-γ, indicating the activation of a proinflammatory phenotype with improved antigen presentation and costimulation capacity [153].

### TAMs are essential for tumor angiogenesis and lymphangiogenesis

TAMs are key cells that control tumor angiogenesis [42, 154] (Fig. 2). Angiogenesis is a crucial process that supplies a growing tumor with nutrients and oxygen. The inhibition of angiogenesis has long been explored as a treatment strategy for colorectal, lung, renal, and cervical cancer and glioblastoma [155, 156]. Widely used FDA-approved antiangiogenic therapy is based on blocking the major proangiogenic factor VEGF or its receptor tyrosine kinases [156]. However, anti-VEGF therapy does not fulfill expectations [157]. The mechanism of therapy resistance in this case can be explained by the activation of alternative angiogenic pathways in response to VEGF blockade [158].

The first study demonstrating the role of TAMs in the angiogenic switch was performed in a mouse model of breast cancer [159]. After that, the ability of TAMs to secrete proangiogenic growth factors (first of all VEGF) and to facilitate the degradation of the perivascular extracellular matrix by a spectrum of released MMPs was shown by multiple studies (summarized in [42]). TAMs are a major source of different types of proangiogenic and extracellular matrix (ECM)-degrading mediators, including VEGF, EGF, PDGF, and TGF-β; angiopoietin 1 and 2 (Ang-1 and -2); matrix metalloproteinases (e.g., MMP2, MMP9, and MMP12); and serine or cysteine proteinases, such as cathepsins and plasminogen activator (PA), which have been identified in both murine models and patient samples [160, 161]. TAMs are also able to release molecules (e.g., TNFa, IL1a, and COX-2) that indirectly contribute to tumor angiogenesis via the induction of a proangiogenic program in tumor cells [160].

Many “nonclassical” growth factors, enzymes, ECM proteins, and other mediators produced by TAMs are involved in the regulation of angiogenesis [42]. These proteins include members of the S100 family, the SEMA family, COX-2, SPP1 (osteopontin), SPARC (osteonectin), Tie-2, and chitinase-like proteins (YKL-39, YKL-40), among others. For example, members of the S100A class (e.g., S100A4, S100A7, S100A8, S100A9, and S100A10) secreted by TAMs induce endothelial cell (EC) proliferation, migration and angiogenesis in vitro and in vivo [162–166].

TAMs are a main source of the chitinase-like protein YKL-39 in breast cancer tissue [167]. YKL-39 combines two activities: it facilitates monocyte recruitment and promotes angiogenesis in vitro [39]. Elevated YKL-39 expression in tumors after neoadjuvant chemotherapy (NAC) is predictive of an increased risk of distant metastasis and a poor response to NAC in patients with breast cancer [39].

Osteopontin (OPN, encoded by SPP1) promotes EC junctional destabilization, actin polymerization and EC motility in vitro and increases microvascular density in vivo [168]. Single-cell RNAseq analysis of colorectal cancer samples revealed that the SPP1-positive population of TAMs was strongly enriched in the tumor angiogenesis, ECM receptor interaction, and tumor vascularisation pathways [89, 169, 170].

TAMs can also express antiangiogenic regulators. For example, SPARC inhibits EC migration and vessel formation in vitro, decreases vessel number, and promotes disruption of the vascular basement membrane in vivo [171]. In our recent study, we demonstrated the clinical value of several angiogenesis regulators produced by TAMs [41]. We analyzed the gene and protein expression levels of S100A4, SPARC and SPP1 in CRC tissue and evaluated their correlations with disease outcome and progression parameters. High S100A4, SPARC and SPP1 mRNA levels were found to be independent prognostic factors for poor survival in CRC patients. Analysis of human CRC tissues revealed that S100A4, SPP1 and SPARC are expressed by stromal compartments, particularly TAMs, and are strongly correlated with macrophage infiltration [41].

The subset of perivascular TAMs (PvTAMs) was thoroughly described by Lewis C. and colleagues in a comprehensive review [172]. In primary tumors, perivascular TAMs mainly express TIE2 and VEGFA and activate leukocyte recruitment and regulation, facilitate the intravasation of tumor cells, promote angiogenesis, and support tumor relapse after chemotherapy. In the metastatic site, the PV macrophage subset, which expresses CCR2 and VEGFA (but not TIE2), promotes cancer cell seeding through direct interactions with cancer cells at the vessel wall and subsequently promotes colonization [172]. Tie2 receptor-expressing TAMs facilitate tube formation and promote EC quiescence and vascular maturation in vitro [173]. Androgen deprivation therapy (ADT) induces the accumulation of TAMs around blood vessels and increases the expression of markers of a protumor phenotype, including folate receptor-beta (FR-β), MRC1 (CD206), CD169 and VISTA, in PvTAMs in human and mouse prostate cancers [174]. In human prostate tumor samples taken before ADT, the density of perivascular FR-β+ CD68+ TAMs was significantly greater in patients who did not respond to ADT than in responders; there was also a nonsignificant trend for PV FR-β+ CD68+ TAMs to also be greater after ADT in nonresponders than in responders [174]. In human invasive breast cancer and MMTV-PyMT tumors, PvTAMs expressing LYVE1 are arranged in close proximity to aSMA+ pericyte-like mesenchymal cells, forming a proangiogenic niche near the vasculature followed by tumor progression [175]. The formation of this niche is dependent on PDGFRα:PDGF-CC cross-talk [175]. LYVE1+ PvTAMs form multicellular nests proximal to blood vessels that are dependent on IL-6-driven CCR5 expression by these TAMs. HO-1 expression on LYVE1+ PvTAMs induces immune exclusion of CD8+ T cells from the TME [176]. PvTAMs can be a transitory subset of CCR2-dependent recruited TAMs that migrate proximally to vessels after 10–14 days of recruitment [177]. The first recruited motile streaming TAMs differentiate into CXCR4-expressing macrophages in a TGF-β-dependent manner and cooperate with CXCL12-expressing cancer-associated fibroblasts in the perivascular niche to promote cancer cell intravasation [177]. Intratumor TAMs located in the tumor parenchyma adopt an mTORC1-low state dependent on tuberous sclerosis complex 1 (TSC1), a negative regulator of mTORC1 signaling [178]. TSC1 deficiency in TAMs reprogrammed them to a pro resolve phenotype with increased mitochondrial respiration, which promoted TAM accumulation in the high-oxygen perivascular region. Perivascular TSC1-deficient TAMs outcompeted PROCR-expressing endothelial cells and suppressed neoangiogenesis, causing tumor tissue hypoxia and starvation-induced cancer cell death [178].

Several factors produced by TAMs are also responsible for the induction of lymphangiogenesis [160]. Among them, VEGFR-3 and its ligands, VEGF-C and VEGF-D, play key roles in lymphangiogenesis [160]. A recent study demonstrated that VEGF-C-expressing TAMs reduce the hematogenous dissemination of mammary cancer cells to the lungs while concurrently increasing lymph node metastasis in a murine breast cancer model [179]. VEGF-C-expressing TAMs express podoplanin and normalize tumor blood vessels [179].

Thus, TAMs control many steps of the angiogenic switch, and to improve the efficacy of currently available antiangiogenic therapies, simultaneous targeting of TAMs is needed [160]. For example, bevacizumab (an anti-VEGF therapy) combined with the CCL2 inhibitor mNOX-E36 decreased the recruitment of TAMs and angiogenesis, resulting in decreased tumor volume in a rat glioblastoma multiforme model [180]. Combined treatment with sorafenib, a small-molecule kinase inhibitor, and TAM depletion with zoledronic acid promoted the inhibition of primary tumor growth and lung metastasis in an orthotopic hepatocellular carcinoma model [181].

### TAMs facilitate tumor cell invasion

The interaction between tumor cells and TAMs contributes to invasion and metastasis, which are the main reasons for the poor prognosis of patients [182]. The metastatic spread of cancer cells starts with their invasion into the bloodstream [182, 183]. To invade, several steps must be completed by cancer cells, including loss of attachment to the surrounding tissue structures, epithelial-mesenchymal transition, ECM degradation, and increased cell motility [143, 184, 185]. TAMs are able to regulate all steps of the metastatic cascade [46]. Secreted by TAMs, IL-1β, IL-8, TNF-α, and TGF-β promote EMT in cancer cells [186, 187]. To initiate ECM degradation, TAMs secrete several proteolytic enzymes, including cathepsins, matrix metalloproteinases (MMPs, such as MMP7, MMP2, and MMP9), and serine proteases [46, 143, 188].

In addition to the functions of TAMs, the contents of cytokines, enzymes, and growth factors and the composition of the ECM in the TME can be defined. The scavenging function of TAMs can control the concentration of growth factors and ECM regulatory components via active scavenger receptor-mediated internalization and degradation [28]. Scavenger receptors (SRs) are a large superfamily of transmembrane proteins with high structural diversity. In the TME, they can recognize and internalize a great variety of ligands, including cytokines, growth factors, modified lipoproteins, and apoptotic cells [28, 189]. The most popular biomarkers of TAMs are scavenger/endocytic receptors [28]. Among them, many international cohorts of cancer patients have shown negative prognostic value for TAMs expressing CD68, CD163, CD204, CD206, MARCO, and stabilin-1 [9, 28]. In several in vitro and in vivo studies, both the pro- and antitumor activities of SRs have been demonstrated to be dependent on the cancer type and type of tumor model [28]. Tumor-supporting activity is related to facilitating tumor invasion, proliferation and migration (mediated by CD204, CD206, CXCL16, stabilin-1, and RAGE), M2-like TAM polarization (by CD36, LOX-1, CXCL16, CD163, and RAGE), and tumor angiogenesis (by CD68, Dectin-1, and RAGE). Tumor-suppressing activity includes the inhibition of tumor angiogenesis (by CD204), tumor invasion (by RAGE), the clearance of tumor cells (by MARCO) and the promotion of M1-like TAM polarization (by CD204 and RAGE) [28]. However, little is known about tumor-related ligands for the SR expressed by TAMs. We identified two tumor-specific ligands of Stabilin-1, SPARC and EGF [33]. In a murine breast cancer model, stabilin-1 was able to promote tumor growth, and this function was linked to the stabilin-1-mediated scavenging of SPARC [33]. The extracellular domains of stabilin-1 can interact not only with SPARC but also with two human chitinase-like proteins, SI-CLP and YKL-39, and this interaction can contribute not only to clearance but also to the intracellular sorting of newly synthesized YKL-39 and SI-CLP and their ability to be secreted [39, 102, 190]. Most recently, we reported that stabilin-1 mediates the clearance of the most potent growth factor that supports the proliferation of cancer cells, EGF [191]. Thus, the cumulative effect of stabilin-1+ TAMs must be considered in a cancer-specific context, and taking stabilin-1 as an example, we suggest that blocking SR function to reprogram TAMs is far from simple and unambiguous, reflected by the absence of advanced clinical trials in this direction (for details, see the last chapter of this review). MARCO is another SR that can carry out some antitumor functions via phagocytosis of tumor cells [192]. However, transcriptomic analysis clearly demonstrated that MARCO is expressed by immunosuppressive TAMs [31, 37]. MARCO targeting has been successful in animal models, where anti-MARCO antibodies block tumor growth and metastasis [95]. However, as we mentioned previously, anti-MARCO antibodies can lead to unfavorable systemic inflammatory complications in patients.

Compared with keratinocyte-derived or melanoma-derived melanosomes, macrophages cografted with fibroblast-derived melanosomes induced enhanced tumor growth and proliferation as well as vascularization in vivo [193]. In vitro, melanoma cells incubated with conditioned media from macrophages loaded with fibroblast-derived melanosomes presented increased proliferation rates and invasive potential. In vitro angiogenesis is induced via the following mechanism: the delivery of AKT1 into macrophages from fibroblast-derived melanosomes activates mTOR phosphorylation, resulting in excessive VEGF secretion [193]. HO-1-expressing TAMs are indicative of tumor invasion and are found at the invasive tumor margin in both human melanoma tumors and a mouse melanoma model in vivo [194]. Myeloid-specific HO-1 deletion in a melanoma model in vivo reduced lung metastasis but did not affect primary tumor growth, indicating that HO-1-expressing TAMs promote metastasis [194].

The next step of the metastatic cascade after matrix remodeling and EMT activation is the invasion of tumor cells into blood vessels [182, 183]. This process is mediated via a paracrine loop involving tumor-synthesized CSF1 and macrophage-produced EGF that drives the migration of tumor cells toward blood vessels [144] (Fig. 2). The direct contact of mammalian‐enabled (MENA)^hi^ tumor cells with perivascular TAMs and endothelial cells at the intravasation site is known as the tumor microenvironment of metastasis (TMEM) [144, 195]. TMEM has been described most thoroughly in breast cancer and represents an independent prognostic indicator of metastatic risk in breast cancer patients [196–198]. The crucial role of the TMEM is VEGFA-dependent disruption of endothelial cell-to-cell adhesions, transient vascular leakiness and tumor cell intravasation [177]. Perivascular TAMs in the TMEM pathway express high levels of the tyrosine kinase receptor TIE2 (also known as CD202b) and CD206 [195]. Perivascular TAMs expressing Tie2 (TEMs) can promote tumor angiogenesis by regulating vascular maintenance (cell proliferation, migration, and stabilization) [42]. TEMs express high levels of other proangiogenic factors, such as MMP-9 and VEGF, and M2-polarize (due to increased levels of COX-2, CD206, and WNT5A) [42, 195]. Hughes R. and others demonstrated that MRC1^+^TIE2^Hi^CXCR4^Hi^ TAMs accumulate around blood vessels in both LLC1 tumors and orthotopic 4T1 and MMTV-PyMT implants after chemotherapeutic impact, as well as in human breast carcinomas after neoadjuvant treatment with paclitaxel [199]. The accumulation of MRC1^+^TIE2^Hi^CXCR4^Hi^ TAMs was accompanied by increased CXCL12 expression in vascularized, well-oxygenated areas after chemotherapy, which was important for tumor revascularization and relapse after chemotherapy. CXCR4 inhibition results in impaired tumor revascularization and regrowth after chemotherapy [199]. Monocytes are recruited to tumor sites via CCR2 signaling, where tumor cell-secreted TGF-β induces CXCR4, stimulating them to migrate toward CXCL12-expressing perivascular cancer-associated fibroblasts (CAFs). In this state, CXCR4-expressing perivascular TAMs can promote cancer cell intravasation [177]. LYVE-1-expressing TAMs have also been characterized recently as perivascular macrophages that create a proangiogenic niche [175, 176].

Once a cancer cell has intravasated into the bloodstream, it becomes a circulating tumor cell (CTC) and can initiate metastasis or be cleared from the blood circulation [200] (Fig. 2). In the bloodstream, CTCs can form clusters with other tumor or nontumor cells, leading to the formation of tumor hybrid cells (THCs). THCs formed by the fusion of CTCs with macrophages exhibit novel properties, including increased proliferation and migration, drug resistance, a decreased apoptosis rate, and the avoidance of immune surveillance [200–202]. In vitro coculture of CTC lines obtained from lung cancer patients with peripheral blood mononuclear cells resulted in the induction of monocyte differentiation into TAMs, which secreted OPN (SPP1), MMP9, chitinase-3-like-1 (YKL-40), and the platelet factor responsible for leukocyte recruitment, migration, and invasion [203]. These macrophage–tumor cell hybrids express M2-like macrophage markers (CD163, CD204, and CD206) and epithelial markers (cytokeratins and EpCAM) and are found in the peripheral blood of patients with PDAC, melanoma, and breast, ovarian, and colorectal cancer [200, 204]. It was also demonstrated that macrophage–tumor cell hybrids are able to promote the formation of metastatic lesions when transplanted into mice, suggesting their role in preparing “niches” for colonization by metastasis-initiating cells [204]. The targeting of TAMs in the context of circulating micrometastasis is highly attractive, primarily because of the low degree of invasiveness of these cells for drug delivery; however, this idea has rarely been explored experimentally.

### TAMs are critical for premetastatic niche formation

TAMs have been suggested to contribute to the formation of premetastatic niches [45, 46, 205–207] (Fig. 2). TAMs themselves are recruited to premetastatic niches by a variety of tumor-secreted factors, such as CCL2, CSF-1, VEGF, PDGF, TNF-α, and TGF-β, where they act in a similar manner as in primary tumors, promoting cancer cell survival [205, 208]. Tumor-derived exosomes that program myeloid cells to be protumoral and proangiogenic can also be important for metastatic niche formation [144, 209]. Exosomes derived from colorectal cancer cells and pancreatic cancer cells that are engulfed by Kupffer cells direct them to initiate favorable premetastatic niche formation in the liver [210, 211]. Metastatic niches are seeded by VEGFR1+ myeloid cells [206]. In spontaneous metastasis models of 4T1 breast cancer and B16F10 melanoma, cytochrome P450 (CYP) 4 A (CYP4A)+ TAMs drive premetastatic niche formation and metastasis development [206]. The pharmacological inhibition of CYP4A reduces lung premetastatic niche formation and the metastatic burden in vivo [206]. In a mouse model of ovarian cancer, CD163+Tim4+ resident macrophages residing in the omentum were shown to be responsible for the metastatic spread of cancer cells [124]. Genetic and pharmacological depletion of CD163+Tim4+ omental macrophages prevents tumor progression and the metastatic spread of disease [124]. The CCL2‒CCR2 axis in the breast tumor microenvironment is crucial for metastasis in the lungs and bones [212, 213]. In lung metastasis of breast cancer, the functional interaction of endothelial cells with perivascular macrophages induces vascular niche formation [214]. Mechanistically, perivascular tenascin C expressed by mammary tumor cells triggered TLR4-dependent activation of perivascular macrophages in the premetastatic niche, which induced the upregulation of *INHBB, LAMA1, SCGB3A1* and *OPG* expression in endothelial cells, which are responsible for metastatic colonization in the lung in vivo. This effect was not suppressed by anti-VEGF therapy; however, combined inhibition of TLR4 and VEGF resulted in more efficient suppression of metastasis than single treatments in vivo [214]. The CXCL10-CXCR3/TLR4 axis is essential for the induction of CCL12 expression in alveolar macrophages in the lung and the formation of the premetastatic niche [215]. Tumor cell-derived CXCL10 increases CCL12 in alveolar macrophages, which leads to the recruitment of monocytic myeloid-derived suppressor cells in premetastatic lungs and the formation of metastases [215]. The formation of a premetastatic niche in the lungs can be dependent on platelet clot formation by tumor cells [216]. Tissue factors derived from tumor cells induce coagulation on tumor cells and then macrophage recruitment. The ability of the clot to recruit CD11b+ macrophages is critical for metastatic cell survival and premetastatic niche establishment in mice [216].

The precise molecular mechanisms of TAM-mediated premetastatic niche formation are still unclear. The role of TAMs in premetastatic niche formation in organs other than the lung needs further investigation.

In summary, this experimental evidence highlights the crucial role of TAMs in all steps of tumor development, beginning with tumor growth support and immune evasion and ending with metastasis formation at distant sites (Fig. 2).

## Molecular pathways that program TAMs and new options for reprogramming

Mechanistically, the protumoral functions of TAMs are programmed at the interface of epigenetic, transcriptional and metabolic events [10]. Macrophages in their advanced polarization state are mostly nondividing cells, and whether the limited proliferation of macrophages significantly contributes to their quantity and differentiation in pathology is still under debate.

Considering that macrophages, in contrast to B cells and T cells, do not undergo genetic rearrangements during their differentiation, the whole spectrum of macrophage subpopulations, as well as their capabilities and limitations in plasticity, are controlled by epigenetic events [217–221]. All levels of epigenetic control, DNA methylation, histone coding, miRNA and long noncoding RNA can contribute to the TAM functional phenotype; however, the speed of response to microenvironment stimuli, as well as reversibility, is much greater at the histone coding and miRNA levels, whereas the stability of programming is the highest at the DNA methylation level.

### DNA methylation in the programming of TAMs

DNA methylation is crucial for monocyte-to-macrophage differentiation [222]. The best-known function of DNA methylation is preventing the transcriptional machinery from assembling on a hypermethylated promoter, resulting in the silencing of gene transcription [223]. Hypermethylation is frequently but not necessarily associated with cell division and is reversible. In cancer cells, DNA methylation is critical for the suppression of the expression of tumor suppressor genes, whereas loss of DNA methylation leads to the overexpression of oncogenes [224]. The effect of tumors on the DNA methylation landscape of TAMs was recently examined in a 4T1 mouse model of triple-negative breast cancer [225]. Compared with those in tumor-bearing mice, the DNA methylation landscapes in macrophages and monocytes from healthy control mice were distinct. Cancer cells significantly change the DNA methylation landscape of macrophages and, to some extent, bone marrow-derived monocytes (BMDMs). The authors were able to link microenvironmental signals to the cancer-specific DNA methylation landscape of TAMs by considering published single-cell transcriptome data, and the integrated approach linked altered cytokine production in the TME and the induction of specific transcription factors linked to the epigenetic reprogramming of TAMs [225]. This study provides a new perspective for the validation of these findings in patient cohorts.

In patients, DNA methylation was suggested to control the expression of interleukin-4-induced-1 (IL4I1, L-phenylalanine oxidase) in M2-like TAMs in human glioma [226]. IL4I1 is associated more frequently than IDO1 or TDO2 with the activity of aryl hydrocarbon receptor (AHR), a ligand-activated transcription factor that can sense tryptophan (Trp) catabolites, enhancing tumor malignancy and suppressing antitumor immunity [227–229]. IL4I1 activated AHR through the generation of indole metabolites and kynurenic acid [228]. Ectopically expressed IL4I1 increased the motility of AHR-proficient but not AHR-deficient cells, and CM from IL4I1-expressing cells reduced T-cell proliferation. The expression levels of IL4I1 are correlated with reduced survival in glioma patients, and high IL4I1-expressing tumors are characterized by an enrichment of suppressive immune cells (MDSCs) and Tregs; however, the major cell types expressing IL4I1 in glioma were not identified in this study [228]. An integrated bioinformatics approach revealed that IL4I1-expressing macrophages in cancer are immunosuppressed by tryptophan degradation, facilitating the recruitment of regulatory T cells into tumors [230]. More recently, reduced methylation of the promoter of IL4I1 was demonstrated to be correlated with aggressive progression and a dismal prognosis for patients with glioma. However, the mechanism that removes methylation from the IL4I1 promoter in TAMs in glioma is unknown [226].

### Histone code in TAM programming

The variability of the enzymatic machinery for both the methylation and demethylation of DNA is significantly limited compared with the broad spectrum of enzymatic machinery that catalyzes a broad spectrum of posttranslational histone tail modifications, making the histone code principal for TAM reactions to the constantly changing tumor microenvironment. Histone modifications, also known as a histone code, are crucial for the high flexibility of macrophages in adjusting their transcriptional mechanisms to the complex and dynamic microenvironment and can be fully explored by cancer cells to program TAMs to support tumor development [10]. The histone code covers a number of posttranslational modifications, such as methylation, acetylation, ubiquitination, citrullination, sumoylation and others, that can modify hotspot amino acids in histone tails [231]. Such modifications can be single, double or triple on the same amino acid and can be homotypic or heterotypic. The sum of the modifications of one amino acid is referred to as a histone mark, and histone marks can be activated, facilitating the relaxation of chromatin, or repressed, stimulating chromatin condensation. The histone code defines a unique functional state of chromatin that regulates various chromatin-templated processes [232]. The sum of histone marks on the promoter defines the level of its availability for the transcriptional machinery and its unique functional state within chromatin. Histone-modifying enzymes can regulate macrophage phenotypes through the addition or removal of functional groups, such as acetyl/methyl groups. Histone modifications are reversible; for example, acetylation and deacetylation are catalyzed by histone acetyltransferases (HATs) and histone deacetylases (HDACs), whereas histone methylation is catalyzed by histone methyltransferases and demethylases, respectively. The variability and reversibility of the histone code and the regulation of the histone-modifying enzymatic machinery ab by stimuli from the microenvironment offer and enable high plasticity for macrophages, including TAMs. Histone acetylation always results in the generation of activating histone marks, while the methylation of histones, depending on the amino acid position and number of methyl groups added, can both be activated and repressed [231]. The histone code acts not only on promoters but also on enhancers that are critical for the differentiation of myeloid precursors, starting from bone marrow progenitors, and for the maturation of macrophages [233, 234]. The best-known activating histone modifications that act on the promoters of genes that control inflammatory programs in macrophages under infectious or metabolic conditions include H3K4me1, H3K4me3, and H3K27ac [235–238]. M2-macrophage marker genes are epigenetically regulated by reciprocal changes in histone H3K4 and H3K27 methylation, and H3K27 is removed by the H3K27 demethylase Jumonji domain containing 3 (Jmjd3). IL-4-dependent Jmjd3 expression was mediated by the interaction of STAT6 with the Jmjd3 promoter. Increased Jmjd3 expression contributes to a decrease in H3K27me2/3 marks as well as the transcriptional activation of M2 marker genes [239]. A recent study applied H3K27ac-ChIP-seq in M2 macrophages and THP-1 cells and revealed that M2-specific enhancers were enriched in Yin Yang, 2 zinc finger nuclear transcription factor (YY1) signals, while YY1 increased macrophage-induced prostate cancer progression by upregulating IL-6 [240]. Histone-modifying enzymes (HMTs, HDMs, HDACs) control the M2 direction of macrophage polarization, which is typical for TAMs, and are of interest for the design of anticancer drugs; however, the typical problem of specific delivery has to be solved to avoid target effects [10]. HDAC2 was shown to regulate the M2-like TAM phenotype via acetylation of histone H3 and the transcription factor SP1 [241]. Suppression of HDAC2 in TAMs suppressed their protumoral secretome, while the spatial proximity of HDAC2-overexpressing M2-like TAMs to cancer cells was significantly correlated with poor overall survival in lung cancer patients [241].

The assembly of the transcriptional machinery on promoters marked by acetylated histones is mediated by bromodomain-containing proteins (BRDs) and some extraterminal motif-containing proteins (BETs) that possess the ability to identify acetylated lysine residues present in histones and other proteins [242]. BRDs and BETs can inhibit or activate the assembly of the transcriptional machinery regulating the production of inflammatory cytokines with crucial functions in tumor progression (IL-1b, IL-6, TNFa, and MCP-1) [10, 243–246].

SWI/SNF chromatin-remodeling complexes, where BRDs (for example, BRD7 and BRD9) are involved, can regulate inflammatory gene expression in macrophages through interactions with lineage-determining and stimulus-regulated transcription factors [247]. Deletion of SWI/SNF subunits in mice resulted in developmental defects in hematopoietic lineages [248, 249]. However, the exact events that can be controlled by BRD and BETs in TAMs remain to be identified. Interestingly, one process that can be affected by BETs is the inhibition of TAMs [250]. NHWD-870, a BRD4 inhibitor that has been reported to be more potent than three major clinical-stage BET inhibitors, BMS-986158, OTX-015, and GSK-525762, blocks the proliferation of TAMs in subcutaneously implanted H526 and A2780 tumors, at least partially by reducing the expression and secretion of CSF1 by cancer cells [250]. Whether NHWD-870 changes the epigenetic landscape in TAMs is the next open question.

### Lactylation of histones as a link between tumor metabolism and TAM programming

Recently, the lactylation of histones in TAMs has attracted the attention of leading research groups working on the epigenetics of TAMs. Lactate is a byproduct of glycolysis and is produced in high amounts by rapidly proliferating cancer cells via aerobic glycolysis. Moreover, the level of lactate can also be elevated under hypoxic conditions, which are typical for rapidly growing tumors. A groundbreaking study by Colegio et al. in 2014 revealed that lactate can induce prohumoral, M2-like programming of tumor-associated macrophages [251]. M2 polarization is mediated by HIF1a but, at least partially, is independent of the IL4-induced pathway [251]. In this study, VEGF and Arg1 were used as read-outs for protumoral M2 polarization of TAMs; however, the downstream events leading to the activation of the promoters of VEGF and Arg1 were not addressed, and only a limited number of flow cytometry-identified parameters of M2 polarization were analyzed.

Transient glycolytic activation of peritumoral monocytes in hepatocellular carcinoma was found to induce sustained expression of carbonic anhydrase XII (CA12) on tumor-infiltrating macrophages via autocrine cytokines and the HIF1α pathway [252]. CA12 mediates the survival of macrophages in acidic tumor microenvironments and stimulates TAMs to produce large amounts of CCL8, which enhances EMT in cancer cells. The accumulation of CA12+ macrophages in the tumor tissues of patients with HCC is associated with increased tumor metastasis and reduced survival in patients with HCC [252]. The fact that transient glycolytic activation in monocytes has a prolonged effect on TAMs can be explained by epigenetic metabolic memory. One mechanism of such memory was identified in human primary monocyte-derived macrophages, where exposure to hyperglycemia resulted in the increased presence of activating histone marks on the promoters of the S100A9 and S100A12 genes [238]. Another potential mechanism of metabolic memory in TAMs is the lactylation of histones.

Histone lactylation was found to be an epigenetic “lactate timer” that switches the acute inflammatory status to the healing program in macrophages [253]. The action of the “lactate timer” can explain why, even in hot tumors, where inflammation can be expected to instruct TAMs toward antitumor activity, there is a failure to reach the level of acute inflammation, corresponding to the levels of the acute antibacterial responses of macrophages. In ovarian cancer, lactate is elevated in the serum of cancer patients and supports tumor growth via the activation of CCL18 expression via H3K18 lactylation in macrophages to promote tumorigenesis [254]. In cancer cells, lactylation of H3K18 can activate nuclear pore membrane protein 121 (POM121), which facilitates the nuclear translocation of MYC, its binding to the CD274 promoter, and the induction of PD-LI expression [255]. Inhibition of glycolysis cooperated with anti-PD-L1 therapy by inducing CD8+ T-cell antitumor effects in mouse NSCLC xenograft models, and elevated levels of overall lysine lactylation (Kla) and specific lactylation of H3K18 (H3K18la) correlated with unfavorable prognosis in NSCLC patients [255]. However, this study did not consider potentially elevated histone lactylation and elevated expression of POM121 in TAMs, and correlation analysis of the elevated levels of Kla and H3K18la performed by single IHC staining did not allow the identification of cell-specific histone modifications. H3K18la was also elevated in epithelial ovarian cancer patient tissues and correlated with poorer OS and PFS; however, the contribution of H3K18la in TAMs remains to be examined [256]. Additionally, the role of H3K18la-mediated elevation of POM121 expression specifically in TAMs cannot be excluded; however, information about the role of POM121 in macrophage activation is extremely limited, but at least one study reported anti-inflammatory effects of POM121 in POM121^fl/fl^ Lyzm-Cre+ mice, which presented elevated levels of lung inflammation after LPS-induced acute lung injury, with increased TNF-α and IL-6 levels in bronchoalveolar lavage fluid (BALF) [257]. The anti-inflammatory effect of POM121 was explained by the inhibition of the NFkappaB pathway in macrophages.

Compared with acetylation, the lactylation of lysines in histones 3 and 4 (H3 and H4) is delayed and is not found on the promoters of acute inflammatory genes but rather on the promoters of genes related to the wound healing activity of macrophages [253]. The wound healing activity of macrophages involves their support of somatic cell migration and proliferation, tissue vascularization, ECM remodeling and suppression of antigen-specific adaptive immunity. These primarily healthy functions of macrophages, which are needed to close wounds, are activated in the tumor microenvironment and are explored by cancer cells to proliferate and enter the vascular system to metastasize. The identification of lactate as a mediator that can change epigenetic programs in macrophages explains why this healing program can never be completed, since lactate is constantly produced by cancer cells. Currently, the candidate lactate writers are p300 (well-known histone acetyltransferase), KAT8 and AARS1 [253, 258]. The overexpression or deletion of p300 in cancer cell lines results in increased or decreased Kla levels, as shown by immunoblotting; however, the effects are not very strong and have not been quantified [253]. Supporting data were produced in murine macrophage-like RAW264.7 cells, where the inhibition of P300 by C646 resulted in a decrease in total protein lactylation levels and increased expression of the inflammatory factors IL-1β, IL-6 and TNF-α [259].

Affinity chromatography, mass spectrometry and in vitro studies in HGC27 cancer cells via loss-of-function and gain-of-function approaches identified alanyl-tRNA synthetase 1 (AARS1), a bona fide lactyl-transferase, which directly uses lactate and ATP to catalyze protein lactylation [258]. AARS1 was found to sense intracellular lactate and translocate it into the nucleus to lactylate and activate the YAP-TEAD complex in HGC27 cells. The authors hypothesized that AARS1, as a Hippo target gene, can form a positive feedback loop with YAP-TEAD, promoting gastric cancer cell proliferation, whereas AARS1 expression is correlated with poor prognosis in GC patients [258]. However, whether AARS1 can act in TAMs has not been investigated. The lysine acetyltransferase KAT8 is a pan-Kla writer on many protein substrates. The interest in KAT8 was due to its interaction with elongation factor 1 alpha (eEF1A2), which was identified via immunoaffinity purification and subsequent LC–MS/MS in eEF1A2-overexpressing HCT116 cells [260]. KAT8 expression was negatively correlated with overall survival (OS) in CRC patients, whereas KAT8 expression was positively correlated with the global Kla level in CRC tissues. Lactylation of eEF1A2K408 supported tumorigenesis via increased protein synthesis, whereas deletion of KAT8 inhibited the growth of colorectal cancer in nude mice injected with HCT116 cells with or without KAT8 depletion. The authors suggested KAT8 as a potential therapeutic target for CRC [260]. However, similar to AARS1, the role of KAT8 in TAMs, which are highly heterogeneous in CRC and can support or inhibit tumor growth, has not been addressed.

The direct cancer-supporting effect of histone lactylation in TAMs was demonstrated in a prostate cancer model of prostate-specific PTEN/p53-deficient genetically engineered mice [261]. Decreased lactate production in PI3Ki-treated cancer cells suppressed histone lactylation (H3K18lac) within TAMs. This promoted TAM phagocytic activity, which was further enhanced by androgen deprivation therapy (ADT) combined with aPD-1 treatment. The cancer-promoting function of histone lactylation in TAMs has been shown in metastatic castration-resistant prostate cancer (mCRPC) patients, whereas single-cell RNA-sequencing analysis of biopsy samples demonstrated a direct correlation between high glycolytic activity and TAM phagocytosis suppression [261]. The same group tested whether the addition of trametinib (MEK inhibitor) to the inhibitor of phosphatidylinositol-3-kinase (PI3K) copanlisib can enhance tumor control in prostate-specific PTEN/p53-deficient genetically engineered mice [262]. They reported an 80% overall response rate via additive suppression of lactate within the TME and histone lactylation at H3K18lac in TAMs relative to monotherapy with copanlisib (37.5%). In resistant mice, Wnt/β-catenin pathway activation via a feedback mechanism results in the restoration of lactate secretion by tumor cells and histone lactylation (H3K18lac) in TAMs. Complete success in 100% of mice over tumor control and activation of TAM antitumor phagocytic activity was achieved when Wnt/β-catenin signaling was suppressed by LGK'974 in combination with PI3Ki/MEKi, which resulted in durable tumor control in 100% of the mice via H3K18lac suppression and complete TAM activation [262].

We are only beginning to identify the potential spectrum of enzymes that catalyze the lactylation and delactylation of histones, but the reversibility of this process is essential for considering such enzymes as therapeutic targets. Even more intriguing is the identification of macrophage-specific writers and erasers of lactylation. Identifying which signaling pathway programming the TAM transcriptome can interact with the histone lactylation process is also highly interesting.

### Interaction between signaling and epigenetic pathways in the protumoral activation of TAMs

Many signaling pathways in TAMs are known to program their protumoral activities (summarized in [10]). Signal transducers and activators of transcription (STATs) constitute a family of transcription factors that were originally identified as classic effectors of interferon-induced signaling and are principally involved in macrophage polarization, where STAT1, in response to IFNγ, induces M1 programming, whereas STAT6, in response to IL4, is responsible for the healing of the M2 phenotype [263–266]. In patients with advanced cervical cancer, an increase in the number of CD68+ pSTAT1+ cells in the tumor mass is correlated with longer disease-free survival (DFS) and overall survival (OS) [266]. In vitro studies and studies in murine models have demonstrated that STAT3, through the polarization of TAMs to the M2 phenotype, facilitates angiogenesis and tumor progression [267–269]. STAT can act cooperatively, and the activation of STAT3 and STAT6 increases cathepsin expression in TAMs, promoting tumor invasion in vivo [264]. IL-4-driven activation of STAT6 inhibited TRIM24 activity, promoting the polarization of macrophages toward the tumor-associated phenotype in a murine model of melanoma [263]. In a murine model of colorectal cancer, activated STAT6 and KLF4 induced M2 polarization of TAMs, leading to tumor progression [265]. TAMs facilitate metastatic colonization via the secretion of IL-35 through the activation of JAK2–STAT6–GATA3 signaling in TAMs, facilitating metastasis in murine mammary carcinoma [270]. This potent role of STATs in M2 polarization resulted in the development of STAT therapeutic inhibitors, and the state of the art for clinical trials is summarized in the last chapter of the review (see Table 3).

A recent study revealed a mechanistic link between the lactylation and activation of STAT3 in tumor-infiltrating myeloid cells (TIMs) [271]. In TIMS, methyltransferase-like 3 (METTL3) mediates m^6^A modification of Jak1 mRNA in TIMs, and the m^6^A-YTHDF1 axis facilitates translation of the JAK1 protein and subsequent STAT3 phosphorylation. Lactate accumulates in the tumor microenvironment, stimulates H3K18 lactylation, and consequently elevates the expression of METTL3 in TIMs. Increased expression of METTL3 in TIMs is correlated with poor prognosis in patients with colon cancer [271]. Thus, targeting STAT3 to improve immunotherapy can be additionally justified by minimizing the protumoral effects of lactate on TAMs [272].

Transcription factors of the nuclear factor-κB (NF-κB) family regulate the expression of genes that control inflammation and immune responses and are pivotal for proinflammatory macrophage programming [273]. Toll-like receptors (TLRs) constitute a major class of drivers of the NF-κB-mediated transcription of inflammatory genes [274, 275]. Thus, targeting TLRs has attracted attention as an easy way to induce inflammation in TAMs (see Table 3). However, inflammation is a double-edged sword in cancer progression [276]. While acute, high-grade inflammation has the potential to kill cancer cells, low-grade inflammation creates optimal conditions at all stages of cancer progression, including initiation, primary tumor growth and metastasis [277–279], and tumor-promoting activation of NF-κB in macrophages has been demonstrated [278, 280]. Although NF-κB is considered a potential activator of the proinflammatory M1 phenotype, the role of NF-κB signaling in TAM plasticity seems to depend on the TME composition of each cancer type. Recently, NF-κB activator 1 downregulation in macrophages was shown to activate STAT3, resulting in immunosuppression and promotion of the transition step from adenoma to adenocarcinoma in colorectal cancer [281]. The link between TLR-mediated pathways and histone lactylation was identified in a mouse model of induced colitis, where macrophage-specific deletion of the TLR signaling adaptor BCAP (B-cell adapter for PI3K) prolonged intestinal inflammation and impaired healing [282]. Mechanistically, the absence of BCAP slowed the inactivation of FOXO1 and GSK3β, increasing inflammation, resulting in defective aerobic glycolysis, and reducing lactate production. The authors concluded that BCAP is a critical switch that facilitates the transition of inflammatory macrophages to healing macrophages by imprinting histone lactylation [282]. These data raise the intriguing questions of how the TLR/BCAP/histone lactylation pathway can program TAMs and whether TLR-mediated NFkappaB activation can contribute to histone lactylation.

There are potential links between the NFkappaB pathway and histone lactylation. In chronic kidney disease, lactate derived from PFKFB3-mediated tubular glycolytic reprogramming enhances histone lactylation, particularly H4K12la, which is enriched at the promoters of NF-kB signaling genes such as Ikb, Rela, and Relb and facilitates the inflammatory response [283]. However, these effects are attributed to the interplay between PFKFB3 and histone lactylation in kidney proximal tubular cells (PTCs), and the role of kidney macrophages has not been addressed. Other evidence for the effect of histone lactylation has already been reported in macrophages, particularly in microglia. Lactic acid levels are significantly elevated in premature microglia, which increases the level of panhistone lysine lactylation (Kla). Both H3K18 lactylation (H3K18la) and Pan-Kla expression were significantly elevated in the senescent microglia and hippocampal tissues of naturally aged mice and AD model mice. H3K18la directly stimulates the NFκB signaling pathway by increasing binding to the promoters of Rela (p65) and NFκB1 (p50), increasing the levels of IL-6 and IL-8 [284]. How histone lactylation affects the NFkappB pathway in TAMs and whether the NFkappaB pathway can regulate histone lactylation enzymes are intriguing open questions.

c-Myc is an essential transcription factor for protumoral TAM programming. c-Myc acts as a clear M2-polarizing transcription factor, is activated by IL-4, and controls the expression of the M2-specific genes SCARB1, ALOX15, and CD206 [285, 286]. Wnt/β-catenin signaling mediates the polarization of M2 macrophages through the activation of c-Myc, facilitating the progression of hepatocellular carcinoma (HCC) [287]. Deletion of c-Myc in TAMs reduced the expression of proangiogenic genes (VEGF, MMP9, and HIF1a) and reduced tumor growth in a mouse melanoma model [288]. In breast cancer cell lines, an increased rate of aerobic glycolysis was shown to support c-Myc expression via the promotion of histone lactylation of its promoter [289], and the possibility that a similar mechanism can act in TAMs cannot be excluded.

The family of interferon regulatory factors (IRFs), which were originally identified as transcription activators and repressors of interferon, also controls macrophage polarization [290]. IRF1, IRF5, and IRF8 contribute to the proinflammatory polarization of macrophages, whereas IRF3 and IRF4 promote M2 polarization in macrophages [291–294]. IRF3 promotes M-CSF-mediated differentiation of monocytes toward M2-type macrophages; inhibits the expression of proinflammatory genes (IL-1α, IL-1β, TNFα, IL-6, IL-8, and CXCL1); and stimulates the expression of protumoral IL-10 and IFN-β [292, 295]. However, TAM polarization to a proinflammatory state can also be dependent on TLR3 and TLR4-IRF3 signaling [296, 297]. IRF3 phosphorylation and transcriptional activity are linked to the TGFbeta pathway and are regulated by Smad2 and Smad3 [298]. IL10 expression in TAMs can also depend on IRF7 [299]. IRF can link signaling and epigenetics in macrophages, and IRF4 and the histone demethylase Jumonji domain containing-3 (Jmjd3) cooperate in the IL-4-induced M2 polarization of macrophages. In contrast, IRF5 activates the expression of inflammatory genes (IL-12p40, IL-12p35 and IL-23p19), promoting M1 polarization [300]. Coexpression of IRF5 and IKKβ (a kinase that phosphorylates and activates IRF5) mediates TAM polarization toward the M1 phenotype, suppressing tumor development in model systems of advanced-stage ovarian cancer, metastatic melanoma, and glioblastoma [294]. In hepatocellular carcinoma cells, IRF5 upregulated the expression of lactate dehydrogenase A (LDHA) and promoted glycolysis, which can further contribute to the inflammatory environment [301]. The outcome of the final outcome of the functional polarization of TAMs will depend on a summary of TF activity and on the metabolic context of the TME. Factors such as IRF5 can actually contribute to the low-grade inflammatory context and increase lactate production, resulting in the most detrimental tumor-supporting mixed M1/M2 phenotype of TAMs.

### Metabolic pathways in TAMs

The clearest biochemical feature that differentiates between M1 and M2 polarization vectors is distinct metabolism. M1 macrophages, which are evolutionarily destined for rapid defense against pathogens, need a shortcut way to energy, which is glycolysis. M1 macrophages utilize highly glycolytic metabolism through the pentose phosphate pathway (PPP) and fatty acid synthesis (FAS), which are pivotal for plasma membrane integrity and reconstitution due to massive receptor turnover and active secretion processes. During active glycolysis and inflammatory signaling, mitochondrial oxidative phosphorylation (OXPHOS) and the tricarboxylic acid (TCA) cycle are impaired in macrophages [302].

In the phase of the resolution of acute inflammation, macrophages switch their metabolism to fatty acid oxidation and use this energy program during healing and in the homeostatic phase after the tissue returns to the normal functional state and healthy turn-over. M2 macrophages are characterized by oxidative metabolism for bioenergetic purposes (OXPHOS), fatty acid oxidation (FAO), decreased glycolysis, decreased metabolism via the PPP and upregulation of arginase 1 (ARG1), which catalyzes the hydrolysis of arginine [303, 304].

The inability of macrophages to switch from glycolysis to fatty acid oxidation keeps them in a state of oscillation between inflammatory and anti-inflammatory signaling, resulting in a chronic inflammatory state. This metabolic indecisiveness is also used by growing tumors to keep TAMs in the “nonending healing” state, which is most beneficial because of the production of a cocktail of cytokines, growth factors and ECM components needed for tumors to expand and invade into vessels. Lipid metabolism in TAMs can also be a target for cancer growth. In a model of murine ovarian cancer, peritoneal macrophages undergo substantial changes in cholesterol metabolism during cancer progression [305]. Confocal microscopy analysis via fluorescently labeled cholera toxin B (CTB, a marker of cholesterol-rich membrane microdomains) revealed that after 21 days of tumor growth, cholesterol-rich microdomains were substantially depleted in peritoneal TAMs. Cancer cells directly drive cholesterol efflux from macrophages and facilitate IL4-driven STAT6-dependent protumor TAM programming, while the deletion of ABC transporters reverts the tumor-promoting programming of TAMs [305]. A novel and conserved TREM2^+^ lipid-associated macrophage (LAM) subset was identified via time-resolved single-cell characterization of the adipose tissue of obese mice [104]. Human TREM2+ LAMs were characterized by further specific gene expression signatures, including LIPA, CTSB, CTSL, FABP4, FABP5, LGALS3, CD9 and CD36. Genetic ablation of Trem2 in mice globally inhibited the downstream molecular LAM program, leading to systemic hypercholesterolemia, body fat accumulation, and glucose intolerance [104]. In triple-negative breast cancer (TNBC), single-cell analysis of TAMs isolated from tumor tissue revealed monocyte-derived STAB1+TREM2high immune suppressive lipid-associated macrophages (LAMs) in patients resistant to immune checkpoint blockade (ICB) [82]. LAM differentiation of monocytes was driven by the CAF-driven CXCL12-CXCR4 pathway. Genetic depletion of this LAM subset in mice suppressed TNBC tumor growth [82].

Therapeutic targeting of TAM metabolic programming is an intensely developing field [306]. There are a number of attempts in preclinical models to target the metabolism of carbohydrates, amino acids, and lipids (summarized in [306]). The metabolism of TAMs can be affected by the microbiota under dietary conditions, which reduces the ability of TAMs to fight tumors. Lactobacillus metabolism of dietary tryptophan to indoles enhances the activity of the aryl hydrocarbon receptor (AhR) in TAMs in pancreatic ductal adenocarcinoma (PDAC) [307]. Deletion of *Ahr* in myeloid cells or pharmacologic inhibition of AhR reduced the growth of PDAC in mice, enhanced the efficacy of PD-L1 blockade, and increased the intratumor accumulation of cytotoxic IFNγ+CD8+ T cells.

Two metabolic inhibitors, RG7356, an inhibitor of CD44, and epacadostat, an inhibitor of IDO, despite promising antitumor effects in preclinical studies, induced severe side effects in patients, and epacadostat failed in phase 3 [308]. The greatest demand for translational oncologists is the delivery of metabolic inhibitors to highly specialized subsets of TAMs, and elegant delivery systems can be designed if two or three determinants on TAMs are considered. Here, scavenger receptors are highly promising because of their expression on M2-TAMs and their intrinsic scavenging activity, which is useful for the internalization of the delivered particles.

## TAMs and anticancer therapy

TAMs can interfere with several therapeutic approaches, including chemotherapy, immunotherapy, radiation therapy, and other targeted therapies [117–119]. The key mechanisms by which chemotherapeutic agents can re-educate TAMs in tumor-protective or antitumor directions include 1) changes in the macrophage phenotype; 2) induced recruitment of monocytes or macrophages to the tumor site; and 3) systemic depletion of monocytes/macrophages [119]. Some chemotherapeutic agents, e.g., doxorubicin, can induce TAM repolarization to the M2 state, induce VEGF production by TAMs and facilitate immunosuppressive macrophage-mediated mechanisms [199]. Platinum-based chemotherapy (cisplatin, carboplatin) supports tumor growth via TAM-secreted exosomes [309]. Paclitaxel protects tumor cells via TAM-produced cathepsins with tumor-protective growth factors [310]. Overall, the data concerning TAM reprogramming under chemotherapy are somewhat contradictory and depend on the type of chemotherapeutic drug, type of cancer, and type of in vitro and in vivo model used. For example, MRC1(+)TIE2(Hi)CXCR4(Hi) macrophages present in human breast carcinomas and bone metastases accumulate around blood vessels in tumors after chemotherapy, where they promote tumor revascularization and relapse [199]. In our recent study, we analyzed the direct effect of the platinum-based chemotherapeutic agent cisplatin on TAM reprogramming in vitro and revealed that the endocytic machinery in TAMs is impaired under cisplatin treatment [191]. We concluded that disrupted scavenging could result in the accumulation of tumor-supporting factors in the tumor microenvironment and therapy resistance [191]. For some cancers, beneficial effects of chemotherapy on TAM programming have been reported. Bulk RNA-seq data of CD45+HLA-DR+Lin−CD14+ cells from ovarian cancer omental metastases taken after neoadjuvant chemotherapy (NAC) revealed increased expression of inflammatory pathways, evidence of inflammasome activation, and a decrease in the tumor-promoting activity of TAMs [311]. CSFR1 inhibitor treatment after chemotherapy significantly decreased disease-free and overall survival in a mouse model, confirming the antitumor role of TAMs after chemotherapy [311]. Single-cell analysis revealed a NACT-induced increase in the expression of HLA class II and antigen-presenting genes in the peripheral blood monocytes of ovarian cancer patients. This effect was accompanied by an increased number of memory T-cell receptor (TCR) clonotypes and an increased number of central memory CD8+ and regulatory T cells after chemotherapy [312]. The prognostic and predictive value of TAMs in patients treated with chemotherapy has been demonstrated [9]. Thus, in colorectal cancer patients treated with 5-fluorouracil, high numbers of TAMs in the invasive front are independently associated with better disease-free survival [313]. Conversely, a high number of CD206+ TAMs and an increase in the CD206/CD68 ratio are correlated with decreased DFS and OS rates after fluorouracil-based chemotherapy in stage II colon cancer patients [36]. In breast cancer patients, high numbers of CD163+ TAMs are correlated with a pathological complete response after neoadjuvant chemotherapy (NACT) [314]. Increased YKL-39 expression after NACT in breast cancer is correlated with a high risk of distant metastasis and a poor response to chemotherapy [39]. In stage II-III lung cancer patients, an elevated CD68+ TAM density in tumors was correlated with superior OS in patients who received NACT [315].

In addition to chemotherapy, immunotherapy has emerged as a standard treatment strategy for cancer [316]. Immune checkpoint inhibitors (ICIs), which include those that target cytotoxic T lymphocyte-associated protein 4 (CTLA-4) and programmed cell death protein 1/programmed cell death ligand 1 (PD-1/PD-L1), have been approved for standard-of-care regimens for patients with several types of tumors [316, 317]. Despite the success of ICIs in multiple clinical trials, only a limited proportion of patients respond to them. All the cellular components of the TME, including TAMs, clearly affect the response to ICIs [72, 73]. For example, the release of the proinflammatory cytokines TNF-ɑ, NFkB and IL-6 by TAMs induces PD-L1 expression in tumor cells via the NF-kB and STAT3 signaling pathways. This mechanism can help tumor cells escape cytotoxic T-cell killing and promote the proliferation of tumor cells [318, 319]. Failure to achieve ICI efficacy may be related to the suppressive interaction between TAMs and T cells. In vitro coculture of TAMs with conventional CD4+ T cells revealed that TAM-derived TGF-β promoted the conversion of conventional CD4+ T cells into immunosuppressive Tregs. Using spontaneous models for breast cancer, it was demonstrated that T-cell conversion was associated with PD-1 expression on intratumoral CD4+ T cells mediated by TAMs [320]. In NSCLC patients, low intratumoral infiltration of CD163+ cells was associated with prolonged PFS and OS during treatment with anti-PD-1/PD-L1 antibodies. In tumors with high macrophage infiltration, the upregulation of genes associated with the IFN-γ signaling pathway and the M1 phenotype was associated with better responses to immunotherapy [321]. The TREM2+ TAM subtype in NSCLC is correlated with an unfavorable prognosis and a low response to PD-1-based therapy [56]. Moreover, TAMs express both the receptor PD-1 and PD-L1 in tumors, which is correlated with M2 polarization. M2 TAMs contribute to ICI resistance by inducing T-cell exclusion and inhibiting T-cell cytotoxic activity. These findings indicate that M2/M1 switching can be a promising strategy for improving tumor immunotherapy [322]. PD-1-expressing TAMs exhibit low phagocytic potential against tumor cells, and blockade of PD-1-PD-L1 increases macrophage phagocytic activity, reduces tumor growth and increases survival in mouse models of cancer [150].

There is still no agreement about the role of TAMs in anticancer treatment response. The results of the use of patient clinical material are contradictory and depend on the patient cohort, type of cancer and type of anticancer drug (Fig. 1). The identification of accurate TAM-based prognostic and predictive biomarkers is complicated by clinical difficulties in identifying tumors before and after treatment. Frequently, authors have reached conclusions on the basis of only the results of either biopsies or tumors after treatment or comparisons of nonpaired samples. This can explain some discrepancies or controversies in the results of different studies. Nevertheless, to achieve maximum treatment efficiency, the molecular mechanisms of the interaction of therapy with TAMs have yet to be established. Deciphering TAM-mediated resistance or interference with already developed immunotherapy tools is urgently needed. The maximal efficiency of immunotherapy can be achieved if the protumor functions of TAMs are blocked. The next chapter summarizes the status of TAM-targeted therapies that are in clinical trials.

## Therapeutic targeting of TAMs

### Targeting CSF1/CSFR1

Since the first identification of the protumor activity of TAMs in the 1970s [323], major discoveries of the mechanism of TAM-mediated cancer support these discoveries in the following decades [324]. The first promising target for suppressing TAM differentiation and accumulation in tumor tissue was CSF1R [325]. CSF1R mediates macrophage differentiation, recruitment and activation via the PI3K-Akt, MEK, PLC, and Erk pathways [326]. Several approaches have been used to target CSF1R signaling, including small molecules that inhibit the tyrosine kinase activity of CSF1R and antibodies that bind CSF1R to block ligand‒receptor interactions or disrupt receptor dimerization [327–329] (Table 1). In 2009, a first-in-class clinical trial was initiated (NCT01004861) using PLX3397 (pexidartinib). Pexidartinib is a receptor tyrosine kinase inhibitor against CSF1R, KIT and FLT3 with IC50 values of 13 nM, 27 nM, and 160 nM, respectively [330]. The first-in-human phase 1 clinical trial in solid tumors revealed good tolerance, pharmacokinetics, and pharmacodynamics (NCT01525602). Pexidartinib was further used to treat several types of cancer, including tenosynovial giant cell tumor (TGCT), prostate cancer, lung cancer, glioblastoma, breast cancer, Hodgkin lymphoma, and acute myeloid leukemia. Some clinical trials have shown benefits for patients, and some are terminated because of limited clinical efficacy (NCT01349036, phase 2 for glioblastoma) [331]. Currently, three clinical trials are ongoing: NCT04488822 (phase 3 for tenosynovial giant cell tumors in China), NCT04703322 (phase 2 for tenosynovial giant cell tumors in Japan), and NCT01042379 (phase 2 for breast cancer). On the basis of the completed phase 3 trial ENLIVEN (NCT02371369), pexidartinib was approved for TGCT treatment by the FDA in 2019. The major compilations of pexidartinib are fatigue, nausea, increased aspartate aminotransferase, increased alanine aminotransferase, periorbital edema and dysgeusia. Other tyrosine kinase inhibitors (TKIs) targeting CSF1R have been developed, and several agents, such as HMPL-012 (surufatinib), DCC-3014 (vimseltinib), JNJ-40346527 (edicotinib), ABSK021 (pimicotinib), CS2164 (chiauranib), and ARRY-382 (PF-07265804), have entered phase 2 clinical trials. HMPL-012 (surufatinib) is a novel tyrosine kinase inhibitor that inhibits CSF1R (IC50: 4 nM), VEGFR1–3 (IC50: 1–24 nM), FGFR1 (IC50: 15 nM), and FLT3 (IC50: 67 nM) [330]. The first-in-human clinical trial was conducted in 2014. On the basis of completed phase 3 clinical trials (NCT02589821, NCT02588170), surufatinib was approved for extrapancreatic neuroendocrine tumors in China in December 2020. It has not received approval from the FDA because of insufficient studies in the United States and further requirements to conduct multiregional clinical trials (https://www.hutch-med.com/suru-fda-nda/). Currently, surufatinib is under investigation in clinical trials for various cancers, including NCT03873532 (phase 2/3, biliary tract cancer), NCT06329947 (phase 2, pancreatic cancer), NCT05668767 (phase 2, small-cell lung cancer), NCT05171439 (phase 2, hepatocellular carcinoma), NCT05236699 (phase 2, cholangiocarcinoma), NCT05106777 (phase 2, osteosarcoma and soft tissue sarcoma), and NCT04764006 (phase 2, colorectal cancer). DCC-3014 (vimseltinib) is another promising agent that potently inhibits CSF1R (IC50 = 2.2 nM) and is being explored in tenosynovial giant cell tumors in phase 3 clinical trials (NCT05059262), Hodgkin lymphoma (NCT05723055, phase 2), and breast cancer (NCT05491226, phase 2). As the company announced in October 2023, vimseltinib met the primary endpoint of a phase III study in patients with TGCTs, as the objective response rate (ORR) reached 40% (95% CI: 29%, 51%) at week 25 (https://investors.deciphera.com/news-releases/news-release-details/deciphera-pharmaceuticals-announces-positive-top-line-results-0). Anti-CSF1R monoclonal antibodies have been proposed for cancer therapy for more than 10 years, and the only one in an active phase III clinical trial is RG7155 (emactuzumab), which has been tested for tenosynovial giant cell tumors (NCT05417789). RG7155 blocks CSF1R dimerization and thus competitively inhibits the binding of CSF1 and IL-34 to CSF1R [332]. RG7155 potently inhibited the viability of CSF-1-differentiated macrophages in vitro and reduced the number of CD68+ CD163+ TAMs in tumor biopsies from diffuse-type giant cell tumor patients [332]. In a phase 1 clinical study (NCT01494688), a dose of 1000 mg every 2 weeks was chosen for the dose-expansion cohort on the basis of pharmacokinetic, pharmacodynamic, and safety information in the dose-escalation cohort. Twenty-four (86%) of 28 patients achieved an objective response, and two (7%) patients achieved a complete response [333]. Common adverse events after treatment were facial edema (16 [64%] of 25 patients), asthenia (14 [56%]), and pruritus (14 [56%]). Five serious adverse events (periorbital edema, lupus erythematosus [occurring twice], erythema, and dermohypodermitis, all experienced by one [4%] patient each) were reported in five patients. FPA008 (cabiralizumab) can bind to CSF1R and competitively inhibits ligand interactions with the receptor [334]. In a phase 1/2 dose escalation and expansion study of cabiralizumab, among the 11 patients treated with 4 mg/kg cabiralizumab, 4 patients achieved PRs (partial response) [335]. Adverse events ≥grade 2 were CK elevation (46%), rash and other skin disorders (36%), fatigue (23%), and periorbital/peripheral/face edema (18%). The use of FPA008 as monotherapy is not attractive, so researchers have attempted to improve therapeutic efficiency via combination therapy with the anti-PD-1 antibody nivolumab. Pancreatic cancer patients were enrolled for combination therapy with FPA008 and nivolumab in a phase 2 clinical trial (NCT03336216). The results did not meet the primary endpoint of progression-free survival (PFS) in 2020 (https://www.targetedonc.com/view/cabiralizumab-misses-primary-end-point-in-phase-ii-trial-of-advanced-pancreatic-cancer), which indicates a barrier for further phase 3 studies. Only a few attempts have been made to target the ligand of CSFR, CSF1, with limited progress to date. MCS110 (Lacnotuzumab) is a humanized monoclonal antibody that binds to CSF1 and blocks downstream signal transduction [336]. MCS 110 was tested as monotherapy in a tenosynovial giant cell tumor (NCT01643850), and the results showed that MCS 110 could shrink tumor sizes. However, the number of patients investigated was small, and further study is needed to determine the efficiency of MCS 110 for TGCTs. A phase 2 clinical trial examined whether combining lacnotuzumab with gem-carbo (gemcitabine plus carboplatin) could improve the outcome of triple-negative breast cancer (TNBC) patients (NCT02435680). The median progression-free survival was 5.6 months [90% CI, 4.47–8.64] in the lacnotuzumab + gem-carbo group and 5.5 months (90% CI, 3.45–7.46) in the gem-carbo group [337]. These findings indicate that the application of lacnotuzumab does not benefit TNBC patients, and no further clinical trials are ongoing. An ongoing study is combining MCS110 with BRAF/MEK inhibitors in phase 2 clinical trials of melanoma patients (NCT03455764).

**Table 1:** Most significant clinical trials targeting CSF1/CSF1R that achieved at least stage 2

Target molecule	Agents, mechanism	Combination with other agents	Cancer types	Starting date of the first clinical trial	ID Clinical trials terminated or failed, stages	ID clinical trials running, stages	In clinics	Complications
**small molecule inhibitor**								
CSF1R	PLX3397 (pexidartinib), TKI (tyrosine kinase inhibitor)	immune checkpoint inhibitor (pembrolizumab), chemotherapy (temozolomide, eribulin)	tenosynovial giant cell tumor, prostate cancer, lung cancer, glioblastoma, breast cancer, Hodgkin lymphoma, acute myeloid leukemia	2009(NCT01004861)	NCT01499043 (phase 2, company decision), NCT01349036 (phase 2, limited clinical efficacy), NCT02452424 (phase 1/2, insufficient clinical efficacy)	NCT04488822 (phase 3), NCT04703322 (phase 2), NCT01042379 (phase 2)	Approved for tenosynovial giant cell tumor (TGCT) treatment by FDA in 2019.	fatigue, liver injury, nausea, dysgeusia, periorbital edema
CSF1R	ARRY-382 (PF-07265804), TKI (tyrosine kinase inhibitor)	immune checkpoint inhibitor (pembrolizumab)	solid tumors, metastatic cancers	2011(NCT01316822)	NCT02880371(phase 1/2, insufficient efficacy, not due to safety reasons)	none	not approved yet	increased transaminases, increased creatine phosphokinase
CSF1R	JNJ-40346527 (edicotinib), TKI (tyrosine kinase inhibitor)	no combination therapy	acute myeloid leukemia, Hodgkin lymphoma, prostate cancer,	2012(NCT01572519)	NCT03557970(phase 2, limited enrollment to determine efficacy)	none	not approved yet	nausea, headache, pyrexia
CSF1R	ARQ087 (derazantinib), TKI (tyrosine kinase inhibitor)	immune checkpoint inhibitor (atezolizumab)	cholangiocarcinoma	2012(NCT01752920)	none	NCT05174650 (phase 2)	not approved yet	fatigue, nausea, increased aminotransferase, diarrhoea
CSF1R	CM082(vorolanib), TKI (tyrosine kinase inhibitor)	mTOR inhibitor(everolimus), immune checkpoint inhibitor (atezolizumab, JS001)	renal cell carcinoma, lung cancer, melanoma	2013(NCT01863485)	NCT03602547 (phase 2, no result posted)	NCT04373369 (phase 2), NCT03848611 (phase 2), NCT03904719 (phase 2), NCT03602547 (phase 2)	not approved yet	neutropenia, leukopenia, increased transaminases
CSF1R	HMPL-012 (surufatinib), TKI (tyrosine kinase inhibitor)	immune checkpoint inhibitor (Camrelizumab, toripalimab, durvalizumab), chemotherapy (paclitaxel, gemcitabine)	neuroendocrine carcinoma, biliary tract cancer, pancreatic cancer, thyroid cancer, lung cancer, breast cancer, colorectal cancer, hepatocellular carcinoma	2014(NCT02133157)	none	NCT03873532 (phase 2/3), NCT06329947 (phase 2), NCT05668767 (phase 2), NCT05171439 (phase 2), NCT05236699 (phase 2), NCT05106777 (phase 2), NCT04764006 (phase 2)	Approved for extrapancreatic neuroendocrine tumors in China in December 2020.	hypertension, proteinuria, hypertriglyceridaemia
CSF1R	CS2164 (chiauranib), TKI (tyrosine kinase inhibitor)	chemotherapy (etoposide, capecitabine, paclitaxel)	lung cancer, ovarian cancer, hepatocellular carcinoma, soft tissue sarcoma	2014(NCT02122809)	none	NCT04830813 (phase 3), NCT04921527 (phase 3), NCT04921527 (phase 3), NCT05497843 (phase 2)	not approved yet	fatigue, proteinuria, hematuria, hypothyroidism, hypertriglyceridemia, hypertension
CSF1R	DCC-3014 (vimseltinib), TKI (tyrosine kinase inhibitor)	immune checkpoint inhibitor (nivolumab, pembrolizumab)	tenosynovial giant cell tumor, Hodgkin lymphoma, breast cancer	2017(NCT03069469)	none	NCT05059262 (phase 3), NCT05723055 (phase 2), NCT05491226 (phase 2)	not approved yet	periorbital edema, fatigue, face edema, pruritus
CSF1R	ABSK021 (Pimicotinib), TKI (tyrosine kinase inhibitor)	no	tenosynovial giant cell tumor, pancreatic cancer	2019(NCT04192344)	none	NCT05804045 (phase 3), NCT06111274 (phase 2)	not approved yet	increased creatine phosphokinase, liver injury
*antibody*								
CSF1R	AMB-05X (AMG-820), inhibits CSF1R interacting with ligand	immune checkpoint inhibitor (pembrolizumab)	tenosynovial giant cell tumor	2011(NCT01444404)	none	NCT05349643 (phase 2)	not approved yet	increased aminotransferase, fatigue, edema, rash
CSF1R	RG7155 (Emactuzumab, RO5509554), anti-CSF1R mAb, inhibits receptor dimerization	anti-VEGF (bevacizumab), chemotherapy (paclitaxel)	tenosynovial giant cell tumor, peritoneal cancer, ovarian cancer	2011(NCT01494688)	NCT02923739(phase 2, terminated by the sponsor)	NCT05417789 (phase 3)	not approved yet	fatigue, facial edema, increased aspartate aminotransferase, increased creatinine phosphokinase
CSF1R	IMC-CS4 (LY3022855), inhibits CSF1R interacting with ligand	immune checkpoint inhibitor (pembrolizumab, tremelimumab, durvalumab), BRAF inhibitor (vemurafenib), MEK inhibitor (cobimetinib)	breast cancer, prostate cancer, melanoma, pancreatic cancer	2011(NCT01346358)	None	NCT03101254 (phase 1/2)	not approved yet	fatigue, nausea, vomiting, diarrhea, anorexia, pyrexia, increased amylase, increased lactate dehydrogenase
CSF1R	FPA008 (Cabiralizumab, BMS-986227), inhibitor of CSF1R interaction with ligand	immune checkpoint inhibitor (nivolumab), chemotherapy (gemcitabine)	pancreatic cancer, biliary tract cancer, peripheral T cell lymphoma, hepatocellular carcinoma	2015(NCT02526017)	NCT03599362 (phase 2, PI departure from institution)	NCT04050462 (phase 2), NCT03697564 (phase 2)	not approved yet	increased creatine phosphokinase, periorbital edema, liver injury
CSF1R	Axatilimab (SNDX6352), inhibitor of CSF1R interacting with ligand	immune checkpoint inhibitor (pembrolizumab, nivolumab, durvalumab), radiotherapy	breast cancer, Hodgkin lymphoma, cholangiocarcinoma	2017(NCT03238027)	none	NCT05491226 (phase 2), NCT05723055 (phase 2)	not approved yet	anemia, nausea, increased aminotransferase
CSF1	MCS110 (Lacnotuzumab), blocks CSF1 binding to receptor	Chemotherapy (carboplatin, gemcitabine), BRAF inhibitor (dabrafenib), MEK inhibitor (trametinib)	breast cancer, prostate cancer, melanoma, synovitis tumor	2009(NCT00757757)	NCT02435680 (phase 2)	NCT03455764 (phase 2)	not approved yet	increased aminotransferase nausea, thrombocytopenia, increased creatinine phosphokinase, fatigue, periorbital edema
CSF1	PD-0360324, blocks CSF1 binding to receptor	no	ovarian cancer, peritoneal tumor, fallopian tube cancer	2015(NCT02554812)	NCT02948101 (phase 2, withdrawn by PI’s request)	none	not approved yet	increased aspartate aminotransferase, fatigue, nausea

In summary, targeting CSF1/CSFR1, when mechanistic efficiency is achieved, has severe side effects on multiple organ and tissue functions because of the pivotal role of monocytes and macrophages in maintaining the immunological integrity of tissue turnover and homeostasis. Therefore, the next idea explored by a number of research groups was not to stop monocyte and macrophage differentiation but to inhibit monocyte recruitment into tumor tissue to reduce the support of cancer cell proliferation and reduce the growth of new vessels needed to provide oxygen and nutrition.

### Targeting the recruitment of monocytes

Chemokines, such as CCL2, CCL5, and CXCL12, are vital for the recruitment of monocytes into tumor tissues [338–341]. At the beginning of the 21st century, CCR2-deficient mice were used to explore the role of CCL2-CCR2 signaling in cervical tumorigenesis [342]. Fewer macrophages are recruited to the cervixes of CCR2-deficient mice. In hepatocellular carcinoma mouse models, the number and size of tumor foci are significantly attenuated in CCR2-deficient mice compared with those in wild-type mice [343]. Disruption of the chemokine‒receptor axis is considered to be a strategy for enhancing cancer therapy [344, 345]. Inhibiting CCL2 suppresses metastatic progression in a mouse model of breast cancer [213] and increases survival in KR158 glioma-bearing mice [346]. Several mAbs and receptor antagonists that target monocyte attractants, such as CCL2, CCL5 and their cognate receptors CCR2 and CCR5 (for summary, see Table 2), have been developed. Despite a number of clinical trials conducted in the past 15 years, chemokine-targeting agents have not achieved clinical application alone or in combination with other therapies. Most agents are trapped in phase 2 studies, and scientists are exploring methods to improve outcomes. Carlumab (CNTO 888), which binds to CCL2 and neutralizes its activity, was the earliest investigated antibody. In a phase 2 clinical trial of prostate cancer patients, no prostate-specific antigen (PSA) or RECIST responses were observed, and the median overall survival (OS) was 10.2 months [347]. Grade ≥3 adverse events occurred in 27 (59%) patients, and 20 (43%) patients experienced serious adverse events, including pneumonia, spinal cord compression, and back pain. Carlumab decreased the concentration of CCL2 in the serum at 24 h postadministration [348, 349]. However, by day 8, the CCL2 concentration had rebounded to pretreatment levels or higher, in some cases reaching threefold greater concentrations than those at baseline. This finding is consistent with the hypothesis that deletion of CCL2 would compensate for the effects of chemokines and result in therapy failure. Plozalizumab (MLN1202) is an anti-CCR2 monoclonal antibody that blocks the CCL2‒CCR2 interaction. In a phase 2 study of bone metastases, urinary n-telopeptide, a biomarker of disease progression, was reduced in 14% of patients, which is insufficient to support further study (NCT01015560). As PD-1 antibodies have been developed in recent years, a phase 1 trial of MLN1202 in combination with nivolumab was initiated (NCT02723006). It was terminated early due to serious adverse events (total 58.33%, including eosinophilia, leukocytosis, acute coronary syndrome, spinal compression fracture and appendicitis), and MLN1202 has been discontinued since then. Small-molecule antagonists against CCR2/CCR5 have been investigated in certain types of cancer, and only BMS-813160 is in a phase 2 study (NCT04123379), which has been applied to non-small cell lung cancer and hepatocellular carcinoma. The CXCL12-CXCR4 axis is another target for inhibiting TAM recruitment. NOX-A12 is an RNA oligonucleotide linked to polyethylene glycol with high affinity for binding to CXCL12 and blocking its interaction with the receptor CXCR4. NOX-A12 has been evaluated for its safety and pharmacokinetics profile and is expected to enroll metastatic pancreatic cancer patients in a phase 2 study (NCT04901741). Several CXCR4 antagonists have been developed, but none are currently in phase 3. Balixafortide (POL6326) is promising but was terminated in a phase III study of breast cancer due to its limited clinical efficacy. BL-8040 (motixafortide) has been evaluated in phase 2 for the treatment of pancreatic cancer (NCT04543071), and plerixafor (AMD3100) has been evaluated in phase 2 for the treatment of glioblastoma (NCT03746080).

**Table 2:** Most significant clinical trials targeting recruitment of TAM that achieved at least stage 2

Target molecule	Agents, mechanism	Combination with other agents	Cancer types	Starting date of the first clinical trial	ID Clinical trials terminated or failed, stages	ID clinical trials running, stages	Complications
CCL2	Carlumab (CNTO 888), binds CCL2 and neutralizes its activity	chemotherapy (docetaxel, gemcitabine, paclitaxel, carboplatin)	prostate cancer, colorectal cancer, pancreatic cancer	2007(NCT00537368)	NCT00992186 (phase 2, no RECIST response)	none	fatigue, nausea
CCR2	Plozalizumab (MLN1202, TAK-202), inhibits CCR2 binding with ligand	immune checkpoint inhibitor (nivolumab)	bone metastases, melanoma	2009(NCT01015560)	NCT02723006 (phase 1, terminated due to adverse events)	none	anemia, increased creatinine, hyperglycemia
CCR2	PF-04136309, inhibits CCR2 binding with ligand	chemotherapy (paclitaxel, gemcitabine)	pancreatic cancer	2011(NCT01413022)	NCT02732938 (phase 2, terminated by business-related decision, not due to safety or efficacy)	none	dysesthesia, diarrhea, hypokalemia, neutropenia, lymphopenia
CCR2	CCX872-B, inhibits CCR2 binding with ligand	no	pancreatic cancer	2015 (NCT02345408)	NCT03778879(Withdrawn because CCX872-B is not available in sufficient quantity to conduct study)	none	diarrhea, neutropenia
CCR2, CCR5	BMS-813160, binds receptors and inhibits activation	immune checkpoint inhibitor (nivolumab), chemotherapy (gemcitabine)	lung cancer, hepatocellular carcinoma, colorectal cancer, pancreatic cancer, renal cell carcinoma	2017 (NCT03184870)	none	NCT04123379 (phase 2), NCT03767582 (phase 1/2)	Diarrhea, Increased amylase, Increased lipase
CCR5	Vicriviroc (MK-7690), inhibits CCR5 binding with ligand	immune checkpoint inhibitor (pembrolizumab)	colorectal cancer	2018 (NCT03631407)	2018(NCT03631407)	none	increased blood bilirubin, Abdominal pain
CCR5	Leronlimab (PRO 140), inhibits CCR5 binding with ligand	TKI (regorafenib), chemotherapy (carboplatin)	colorectal cancer, breast cancer,	2019 (NCT03838367)	none	NCT04504942 (phase 2)	lymphopenia
CXCL12	NOX-A12, binds CXCL12 and neutralizes its activity	chemotherapy (bendamustine)	chronic lymphocytic leukemia (CLL), glioblastoma, multiple myeloma, pancreatic cancer, colorectal cancer	2011 (NCT01486797)	none	NCT04901741 (phase 2)	neutropenia
CXCR4	Balixafortide (POL6326), inhibits CXCR4 binding with ligand	chemotherapy (eribulin)	breast cancer	2013 (NCT01837095)	NCT03786094(phase 3, terminated due to failure to meet the primary endpoint)	none	Neutropenia, Diarrhoea, Nausea
CXCR4	BL-8040 (Motixafortide), inhibits CXCR4 binding with ligand	chemotherapy (gemcitabine, paclitaxel, cytarabine), immune checkpoint inhibitor (cemiplimab, atezolizumab)	chronic myeloid leukemia, acute myeloid leukemia, multiple myeloma, gastric cancer, pancreatic cancer,	2014(NCT02115672)	none	NCT04543071 (phase 2)	neutropenia, Thrombocytopenia, Constipation, Diarrhea, Anemia
CXCR4	plerixafor (AMD3100), inhibits CXCR4 binding with ligand	immune checkpoint inhibitor (pembrolizumab, cemiplimab), chemotherapy (temozolomide), radiotherapy	pancreatic cancer, head and neck squamous cell carcinoma, pancreatic cancer, ovarian cancer, colorectal cancer, glioblastoma	2014(NCT02179970)	NCT04058145(phase 2, withdrawn due to funding)	NCT03746080 (phase 2)	Thrombocytopenia, Diarrhea
CXCR4	LY2510924, inhibits CXCR4 binding with ligand	immune checkpoint inhibitor (durvalumab), TKI (sunitinib), chemotherapy (carboplatin, etoposide)	acute myeloid leukemia, renal cell carcinoma, lung cancer	2016(NCT02652871)	NCT01391130 (phase 2, terminated due to insufficient efficacy)	none	anemia, neutropenia, leukopenia, vomiting

Thus, the importance of monocyte recruitment into damaged or transformed tissue is essential for the functioning of the body, and this process can be evolutionarily ensured by the number of compensatory mechanisms through which CCR2 or CCL2 cannot perform their functions. This approach has been used not only for cancer but also for cardiovascular disorders, diabetic kidney disease, pulmonary fibrosis, osteoarthritis, multiple sclerosis and other diseases without providing an approved drug [350]. The complications observed in these patients are neutropenia, thrombocytopenia, and diarrhea, which are similar to the complications reported in cancer patients. Thus, the promise of targeting monocyte recruitment, at least as a monotherapy approach, has significant limitations.

### Reprogramming TAMs

Reprogramming TAMs is proposed to be an effective strategy to fight against tumors, including blocking protumor activity and activating antitumor activity [325, 351]. The NFkappaB pathway was one of the first suggested targets for re-education of tumor-promoting TAMs, and promising results were generated in murine cancer models [278]. STAT3 is a fundamental transcription factor for macrophage polarization, acting downstream of Janus kinase (JAK) signaling [352, 353]. Small-molecule inhibitors of STAT3 have been shown to act via different mechanisms. OPB-31121 inhibits the phosphorylation of STAT3 and subsequently suppresses target promoters [354–356]. It was well tolerated by hepatocellular carcinoma patients in a phase 1/2 study (NCT01406574); however, no further information about the continuation of the clinical trials for OPB-31121 is available. Another STAT3 inhibitor is WP1066, which blocks the translocation of p-STAT3 and inhibits STAT3-mediated transcription activation. It is under evaluation in a phase 2 clinical trial for glioblastoma patients in combination with radiation therapy (NCT05879250). Toll-like receptors (TLRs) are abundantly expressed in macrophages, and the activation of TLRs is an attractive way to convert TAMs into the M1-like phenotype [357, 358]. NPs loaded with vR848 (an agonist of the toll-like receptors TLR7 and TLR8) were delivered to tumor tissues in an immunocompetent mouse model of colorectal cancer (MC38) in C57BL/6 mice and promoted the M1 phenotype of TAMs as well as suppressed carcinogenesis [359]. RAW264.7 cells were first stimulated with LPS and Ac_4_ManNAz for M1 polarization and then anchored with liposomes containing a TLR7/8 agonist [360]. The engineered macrophages (LAMΦ-m7/8a) phagocytosed 4T1 tumor cells in vitro and expressed high levels of IL-6 and TNFa for 48 h in vitro. Both intratumoral and intravenous injections of LAMΦ-m7/8a decreased the tumor burden in combination with doxorubicin-loaded liposomes in 4T1-tumor-bearing mice. Increased infiltration of CD8 + T cells and reduced numbers of myeloid-derived suppressor cells were observed in the TME. The TLR7/8 agonist imiquimod was constructed into nanozymes and induced the expression of proinflammatory cytokines, including TNF-α and IL-6, in RAW 264.7 cells [361]. The nanozymes increased the ability of RAW 264.7 macrophages to phagocytose U2OS cancer cells in a coculture experiment (no tumor-bearing mouse model was used in this study). The TLR9 agonist ODN1826 suppressed tumor burden in a B16-F10 melanoma mouse model, and the most efficient treatment was the locally injected combination of the CpG oligonucleotide TLR9 agonist ODN1826 combined with systemic CTLA-4 blockade [362]. Agonists for TLRs (TLR3, TLR4, TLR7/8, and TLR9) have been investigated in clinical trials as agents for tumor therapy (Table 3). The TLR3 agonist Rintatolimod is intravenously injected into pancreatic cancer patients in a phase 2 clinical trial (NCT05494697), which is recruiting patients. Considering the potential systemic toxicity of TLR activation, intratumoral or subcutaneous administration of TLR-targeting agents has been applied in some clinical trials. CMP-001, a TLR9 agonist, is injected intratumorally into melanoma patients (NCT03618641, phase 2), and the results revealed that 46.7% (14/30) of patients achieved a partial response. The subsequent phase 2/3 study (NCT05059522) for melanoma is ongoing. BO-112, a TLR3 agonist, is used intratumorally in combination with intravenous pembrolizumab to treat melanoma patients (NCT04570332, phase 2). The TLR8 agonist motolimod (VTX-2337) was subcutaneously administered in a phase 2 study (NCT01666444). The results revealed that the addition of motolimod to pegylated liposomal doxorubicin did not improve overall survival or progression-free survival [363]. The TLR7 agonist TMX-101 was intravesically administered for bladder cancer. A phase 2 study [364] revealed that 20% (2/10) of patients demonstrated negative (tumor-free) cytology and biopsy results after 6 weeks of treatment. However, the number of patients is limited, and larger cohort studies are needed to validate the therapeutic efficacy of TMX-101.

**Table 3:** Most significant clinical trials targeting TAM activity that achieved at least stage 2

Target molecule	Agents, mechanism	Combination with other agents	Cancer types	Starting date of the first clinical trial	ID Clinical trials terminated or failed, stages	ID clinical trials running, stages	Complications
***Blocking of pro-tumor activity***							
STAT3	OPB-31121, inhibits phosphorylation of STAT3	no	Non-Hodgkin’s Lymphoma(NHL), multiple myeloma, hepatocellular carcinoma	2007(NCT00511082)	NCT01406574 (phase 1/2)	none	nausea, vomiting, diarrhea
STAT3	WP1066, blocks nuclear translocation of p-STAT	radiotherapy	glioma, melanoma	2013(NCT01904123)	none	NCT05879250 (phase 2)	Diarrhea, leukopenia
*Activation of anti-tumor activity*							
TLR8	Motolimod (VTX-2337), binds and activates TLR8	chemotherapy (doxorubicin, carboplatin, cisplatin)	ovarian cancer, head and neck squamous cell	2008(NCT00688415)	NCT01666444 (phase 2)	none	diarrhea, vomiting, neutropenia
TLR7	TMX-101, binds and activates TLR7	no	bladder cancer	2010(10.1016/j.juro.2012.11.150)	NCT01731652 (phase 2)	none	micturition urgency, dysuria (intravesical administration)
TLR3	Rintatolimod, binds and activates TLR3	chemotherapy(oxaliplatin, irinotecan)	colorectal cancer, breast cancer, ovarian cancer, pancreatic cancer	2011(NCT01355393)	none	NCT05494697(phase 2)	increased aminotransferase, anemia, nausea, vomiting, neutropenia
TLR3	BO-112, binds and activates TLR3	immune checkpoint inhibitor (pembrolizumab)	lung cancer, melanoma	2016 (NCT02828098)	none	NCT04570332 (phase 2)	(minimal systematic side effect as intra-tumoral administration)
TLR9	CMP-001 (vidutolimod), binds and activates TLR9	immune checkpoint inhibitor (avelumab)	melanoma, ovarian cancer, lung cancer, basal cell carcinoma, prostate cancer, breast cancer	2016 (NCT02680184)	none	NCT05059522(phase 3), NCT04916002(phase 2), NCT05445609(phase 2), NCT04807192(phase 2)	hyperuricemia, hypoalbuminemia, neutropenia, nausea
CD47	Hu5F9-G4 (Magrolimab), binds to CD47 and blocks interaction with SIRPα	immune checkpoint inhibitor (pembrolizumab), chemotherapy (paclitaxel, azacitidine)	colorectal cancer, ovarian cancer, breast cancer, prostate cancer, acute myeloid leukemia, myelodysplastic syndrome(MDS), Hodgkin lymphoma	2014(NCT02216409)	NCT05079230 (phase 3) (February 2024, FDA halts clinical studies of magrolimab in AML, MDS, because the agent is futile and increased the risk of death in this population), NCT04313881(phase 3, futility)	NCT04958785(phase 2), NCT05330429(phase 2), NCT04854499(phase 2)	constipation, nausea, diarrhea, anemia
CD47	lemzoparlimab (TJ011133), binds to CD47 and blocks interaction with SIRPα	chemotherapy (azacitidine)	acute myeloid leukemia (AML), myelodysplastic syndromes(MDS)	2019(NCT03934814)	NCT04912063 (phase 1, terminated due to company decision)	NCT05709093(phase 3)	nausea, diarrhea, leukopenia
CD47	AK117, binds to CD47 and blocks interaction with SIRPα	Chemotherapy (paclitaxel, Azacitidine, Oxaliplatin)	breast cancer, colorectal cancer, gastric cancer, acute myeloid leukemia, myelodysplastic syndrome,	2020(NCT04349969)	none	NCT05227664(phase 2), NCT05382442(phase 2), NCT05960955(phase 2), NCT06196203(phase 2),	nausea, diarrhea
CD47	TTI-621 (Trillium), SIRPα fusion protein, acts as CD47 antagonist	immune checkpoint inhibitor (pembrolizumab)	multiple myeloma, diffuse large B-Cell lymphoma, leiomyosarcoma	2016(NCT02663518)	NCT04996004 (company’s decision, not due to safety concerns or authority request)	NCT05507541(phase 2)	thrombocytopenia, anemia, neutropenia, leukopenia
CD47	Evorpacept (ALX-148), SIRPα fusion protein, acts as CD47 antagonist	immune checkpoint inhibitor (pembrolizumab), chemotherapy (doxorubicin)	colorectal cancer, ovarian cancer, non-hodgkin lymphoma, gastric cancer, head and neck cancer, breast cancer	2017(NCT03013218)	none	NCT05002127 (phase 2/3) NCT05167409 (phase 2), NCT05467670 (phase 2), NCT05787639 (phase 2), NCT04675294 (phase 2)	increased AST, platelets decreased
CD47	IMM01, SIRPα fusion protein, acts as CD47 antagonist	chemotherapy (azacitidine), immune checkpoint inhibitor (tislelizumab)	Hodgkin lymphoma, acute myeloid leukemia, myelodysplastic syndromes	2021(NCT05140811)	none	NCT05833984(phase 1/2)	No available information
CD47, PD-L1	IBI-322, anti-CD47/anti-PD-L1 bispecific antibody	TKI (lenvatinib)	lung cancer, hematologic malignancy	2020 (NCT04328831)	none	NCT05296603 (phase 2)	No available information
CD47, CD20	IMM0306, anti-CD47/CD20 bispecific antibody	no	B-cell Non-Hodgkin’s Lymphoma	2021 (NCT04746131)	none	NCT05805943(phase 1/2)	No available information
CD40	APX005, binds and activates CD40	Chemotherapy (paclitaxel), immune checkpoint inhibitor (nivolumab), radiotherapy	glioblastoma, lung cancer, melanoma, pancreatic cancer	2015 (NCT02482168)	NCT04337931(phase 2, company’s decision, not due to safety reason)	NCT04130854 (phase 2)	pyrexia, chills, nausea, fatigue, pruritus, elevated liver function, rash, vomiting
CD40	SEA-CD40, binds and activates CD40	immune checkpoint inhibitor (pembrolizumab), chemotherapy (pemetrexed, carboplatin)	melanoma, lung cancer	2015 (NCT02376699)	none	NCT04993677 (phase 2)	nausea, neutropenia, anemia, thrombocytopenia
CD40	CDX-1140, binds and activates CD40	immune checkpoint inhibitor (pembrolizumab), radiotherapy, chemotherapy(gemcitabine, paclitaxel)	breast cancer, pancreatic cancer, ovarian cancer	2017 (NCT03329950)	NCT04536077 (phase 2, company’s decision)	NCT05231122(phase 2)	arthralgia, nausea, diarrhea, vomiting, increased AST, increased bilirubin

### Targeting CD47/SIRPα

Tumor cells express the “do not eat me” signal CD47 to interact with SIRPα on macrophages, which contributes to escape immune elimination [365–367]. Interference with CD47/SIRPα can restore the phagocytic activity of TAMs toward cancer cells. Several antibodies have been developed to target CD47, which blocks the activation of SIRPα and reprograms TAMs to engulf tumor cells [368, 369] (Table 3). Hu5F9-G4 (megrolimab) is the first-in-class anti-CD47 antibody and has passed through a phase 1 clinical trial for the treatment of ovarian cancer, colorectal cancer, prostate cancer and hematological malignancies. Phase 2 clinical trials in several solid cancers, including breast cancer (NCT04958785), colorectal cancer (NCT05330429), and head and neck squamous cell carcinoma (NCT04854499), are ongoing. In February 2024, the FDA halted clinical studies of magrolimab in acute myeloid leukemia and myelodysplastic syndrome because the agent is futile and increases the risk of death in these patients, probably resulting in the discontinuation of phase 3 studies (NCT05079230). Lemzoparlimab (TJ011133) is another anti-CD47 antibody that is used to treat myelodysplastic syndrome patients in phase 3 studies (NCT05709093). The SIRPα fusion protein is composed of a modified SIRPα domain and an Fc region of human immunoglobulin G, which maintains the ability to bind CD47 and to abrogate the CD47/SIRPa-mediated inhibition of macrophage phagocytosis [370]. The most advanced version of the therapeutic SIRPα fusion protein is evorpacept (ALX-148), for which phase 2/3 clinical trials were initiated for patients with HER2^+^ gastric cancer (NCT05002127). The company ALX Oncology also conducts phase 2 studies on colorectal cancer (NCT05167409), ovarian cancer (NCT05467670), oropharynx cancer (NCT05787639), and head and neck squamous cell carcinoma (NCT04675294). As immunotherapies for cancer therapy and combination treatment are attempting to improve outcomes, TTI-621 (Trillium) has been evaluated in diffuse large B-cell lymphoma in combination with the anti-PD-1 antibody pembrolizumab (NCT05507541). The development of bispecific antibodies (bsAbs) for cancer therapy is also promising. One type of such bispecific antibody platform is based on one arm recognizing CD47, while the other arm recognizes a cell type-specific target. The anti-CD47/anti-PD-L1 bispecific antibody IBI-322 entered a clinical study in 2020 and is currently in phase 2 for small-cell lung cancer (NCT05296603).

### Targeting CD40

CD40 has attracted much attention as a cancer therapy target [325, 345]. CD40 is a member of the tumor necrosis factor (TNF) receptor superfamily and is expressed by antigen-presenting cells (APCs), including macrophages [371, 372]. When activated, CD40 triggers intracellular signal transduction to release proinflammatory cytokines, including NF-KappaB, MAPK and STAT3 [324, 373, 374]. An agonist CD40 antibody was tested in a mouse model of pancreatic ductal adenocarcinoma, and the results revealed that it suppressed tumor progression [375]. Several anti-CD40 agonist mAbs, including LIST, with a high affinity for binding to CD40 and priming for macrophage activation have been developed [376, 377]. Currently, phase 2 clinical trials are ongoing for APX005M for rectal cancer (NCT04130854), SEA-CD40 for non-small cell lung cancer and melanoma (NCT04993677), and CDX-1140 for ovarian cancer (NCT05231122). It is under investigation whether CD40-targeting therapy can improve the outcomes of cancer patients. In addition to being expressed by TAMs, CD40 is expressed on multiple immune cells, vascular cells, neurons, and other somatic cells. Therefore, the use of CD40 agonists can reduce the antimicrobial activity of the immune system, impair healing and increase the risk of thrombosis and cardiovascular complications [378–381].

### Modulation of TAM metabolism

Accumulating data demonstrate that metabolic pathways can modulate the function of macrophages, including glucose, the TCA cycle, fatty acids, and amino acids [139, 306, 372, 382]. GLUT1 was increased in the TAMs of PDAC human samples, and a higher proportion of GLUT1-positive cells was correlated with poorer survival [383]. GLUT cKO mice displayed reduced tumor growth, and the GLUT1 inhibitor WZB117 attenuated the tumor burden in vivo. LDHA cKO mice exhibited reduced lung cancer, decreased angiogenesis and an increased antitumor CD8+ T-cell response [384]. The plasticity of TAMs allows metabolic intervention to reshape their phenotype, which has become a novel therapeutic strategy [385, 386]. Preclinical studies in mice have revealed the potential of targeting TAM metabolism for tumor therapy. Etomoxir (an inhibitor of fatty acid oxidation) can inhibit 5TGM1 myeloma tumor growth in mouse models [387]. CD44 is a transmembrane glycoprotein exposed on the cell surface. Previous studies have shown that CD44 modulates monocyte differentiation via the phosphorylation of ERK1/2 [388]. RG7356, an anti-CD44 humanized antibody, was well tolerated in a phase 1 clinical trial [389], but whether a phase 2 clinical trial is going to be conducted has not yet been announced. Indoleamine-2,3 dioxygenase 1 (IDO1) is a regulator of immune cells. Encouraged by early phase trials, a phase 3 study (ECHO-301/KN-252) was launched in metastatic melanoma to evaluate the combination of epacadostat and pembrolizumab. However, no significant differences were found between the epacadostat group and the placebo group in terms of either progression-free survival or overall survival [390]. More investigations are needed to explore the potential and efficiency of metabolism-targeting strategies for cancer therapy.

### Monocytes and macrophages as cell therapy tools

The first attempts to use programmed monocytes to rate cancer incidence were reported in the 1990s [391, 392]. In the laboratory of R Andreesen, peripheral blood monocytes were used to generate macrophages after treatment with GM-CSF [393]. Monocyte-derived macrophages were transferred into 11 patients with melanoma and 1 patient with renal cell cancer. No objective clinical response of the tumor was observed, and no dose-limiting toxicity was observed. It was concluded that ex vivo programming of macrophages is insufficient to treat tumors. Several years later, macrophage plasticity was described and demonstrated by several studies [394–396], which explained the failure of cytokine-programmed monocytes/macrophages in cancer therapy. Most recently, a genetically engineered approach was developed to fix the antitumor functions of macrophages. such as CAR-macrophages [397] (Table 4).

**Table 4:** Pre-clinical and clinical studies on monocytes and macrophages as cell therapy tools for cancer

cell types	modification	administration	cancer type	combination therapy	study date	current stage	mechanism
autologous peripheral blood mononuclear cells (PBMCs)	transfected by flow electroporation with mRNA encoding anti-mesothelin chimeric antigen receptor (CAR)	intraperitoneal	Adenocarcinoma of Ovary, Peritoneum or Fallopian Tube; Peritoneal Mesothelioma	no combination	2018	phase 1, terminated because sponsor shifted focus (NCT03608618)	CAR-transfected PBMCs recognize, bind to, and induce phagocytosis of cancer cells expressing mesothelin. In addition, MCY-M11 stimulates immune system to induce a cytotoxic T-lymphocyte response against mesothelin-expressing cancer cells
CD34+ hematopoietic stem/progenitor cells	transduced with a lentiviral vector expressing IFNα2	intravenous	Glioblastoma	no combination	2019	Phase 1/2 ongoing (NCT03866109)	release of IFN-α inside tumors and inducing anti-tumor immune response
induced pluripotent stem cells	transduced with lentiviral vector to express CD19-CAR or mesothelin-CAR	intraperitoneal	ovarian cancer	no combination	2020	preclinical mice model (DOI: 10.1186/s13045-020-00983-2)	promoting macrophages polarization toward anti-tumor phenotype and enhancement of phagocytosis of cancer cells
hematopoietic stem/progenitor cells	transduced with pELNS lentiviral vector to encode p35 and p40 subunits of IL-12	intravenous	lung metastasis	no combination	2021	preclinical mice model (doi: 10.1016/j.cell.2021.02.048.)	genetically engineered myeloid cells (GEMys) express high levels of IL-12 in tumor tissues and reverses immune suppression in the pre-metastatic niche by activating antigen presentation and T cell
autologous monocyte-derived macrophages	transduced with adenoviral vector Ad5f35 to express a CAR targeting HER2	intravenous	HER2-positive solid tumor	monotherapy, in combination with pembrolizumab	2021	phase 1 ongoing (NCT04660929)	anti-HER2 CAR Macrophages recognize and bind to HER2-expressing tumor cells, resulting in phagocytosis. Also, maintain anti-tumor M1 phenotype within tumor microenvironment
induced pluripotent stem cell-derived macrophages	transduced with lentiviral vector to express CARs containing CD3ζ and toll-like receptor 4 intracellular toll/IL-1R (TIR) domain	intraperitoneal	hepatocellular carcinoma	no combination	2023	preclinical mice model (DOI: 10.1038/s41590-023-01687-8)	CD3ζ-TIR dual signaling CAR increases engulfment capacity and M1 polarization
pluripotent stem cells	integrating an anti-GD2 CAR into the AAVS1 locus by CRISPR-Cas9 gene editing method	subcutaneous	neuroblastoma	no combination	2023	preclinical mice model (DOI: 10.1016/j.stemcr.2022.12.012)	phagocytosis of GD2-expressing neuroblastoma and melanoma in vitro, activation of antigen processing
none	in situ injection of hydrogel containing nanoparticles of CD133-CAR plasmids (pCARs)	intracavity	glioblastoma	no combination	2022	preclinical mice model (DOI: 10.1126/scitranslmed.abn1128)	reprogramming an intracavitary macrophages’ phagocytic function against tumor
none	intravenous infusion of lentiviral vectors with coding sequence of IFNa	intravenous	liver metastasis	no combination	2023	precinical mice model (DOI: 10.1016/j.ccell.2023.09.014)	activation of liver macrophages in vivo, including Kupffer cells and TAMs, which enhanced antigen presentation and promoted CD8 + T cell effector function
none	in situ programming of myeloid cells by lipid nanoparticles with mRNA encoding CAR	intravenous	epithelial cancer	no combination	2023	phase 1 ongoing (NCT05969041)	mRNA of TROP2-targeting chimeric antigen receptors is delivered and expressed in myeloid cells, inducing anti-tumor effect against TROP2+ tumor cells

**Fig. 3:**
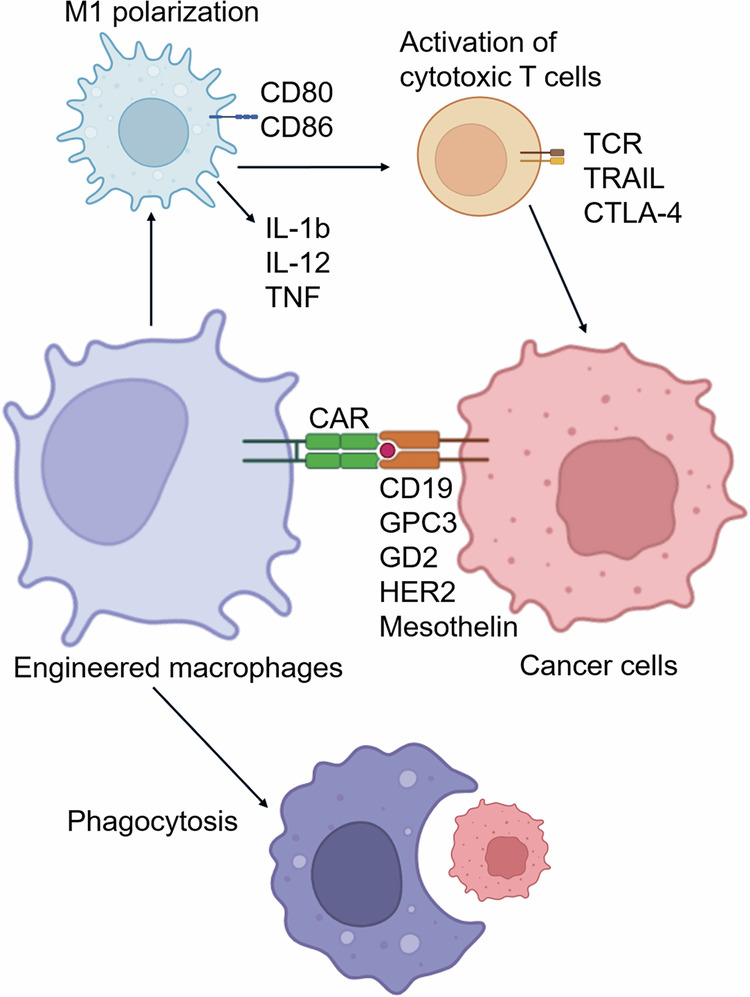
Mechanisms of CAR-macrophage-mediated targeting of cancer cells. CAR-T cells recognize and bind to target molecules on the cancer cell surface, that triggers intracellular signaling, including NF-kB, STAT3, and JAK. The primary effect of CAR-T cells is phagocytosis of cancer cells and their elimination. The additional effect is M1 polarization of TAMs, which involves elevated expression of CD80 and CD86 and increased secretion of proinflammatory cytokines, stimulating the cytotoxic T-cell response

Genetically engineered macrophages target several essential factors for tumor control activities: phagocytosis, cytokine secretion critical for adaptive cytotoxic responses, and modifications of the TME facilitating metastasis (reviewed in [398]). CAR-CMs are modified with the desired antigen-specific chimeric receptor (CAR) on the cell surface and are evaluated as therapeutic tools for solid tumors [399] (Fig. 3). The CAR consists of an extracellular antigen recognition domain, a hinge domain, a transmembrane domain, and one or more cytoplasmic signal domains [400]. Depending on the antigen recognition domain, CAR-T cells recognize target cells and induce a proinflammatory immune response [401]. CAR-iPSCs were developed from induced pluripotent stem cells by transduction with lentiviral vectors and culture in differentiation medium [402]. CAR (CD19)-iMacs, which are cell lines that overexpress CD19, exhibit increased phagocytosis activity against K562 leukemia cells. CAR (meso)-iMacs exhibited increased phagocytic activity against OVCAR3 and ASPC1 cells, which are ovarian and pancreatic cancer cells that overexpress mesothelin. CAR (meso)-iMac suppressed ovarian cancer progression in a mouse model. Induced pluripotent stem cell-derived mouse macrophages were engineered into CAR-CMs with Toll-like receptor 4 (TLR4) intracellular toll/IL-1R (TIR) domain-containing CARs [403]. The CAR-iMACs exhibited an M1-like phenotype, and both the TIR and CD3ζ domains contributed to the phagocytosis of U87MG cancer cells. CAR-iMACs displayed a significant antitumor effect on a hepatocellular carcinoma (HCC) mouse model of HepG2 cells. The CRISPR-Cas9 gene editing method was used to integrate an anti-GD2 CAR into mouse pluripotent stem cells, and these cells were differentiated into macrophages in culture medium, which were named anti-GD2 CAR macrophages [404]. Anti-GD2 CAR macrophages phagocytosed GD2-expressing neuroblastoma and melanoma cells in vitro and suppressed neuroblastoma tumor growth in a mouse model. Hematopoietic progenitors of mice were transduced with a Tie2 promoter/enhancer-driven Ifna1 gene and turned into Tie2-expressing monocytes [405]. An in vivo mouse study revealed that these Tie2-expressing monocytes efficiently increase IFNa levels in tumor tissues and suppress tumor burden in gliomas and mammary carcinomas. Encouraged by preclinical studies [405, 406], the cell therapy tool Temferon was developed from hematopoietic stem/progenitor cells and expresses IFNα2 (Genenta Science. Inc.). The phase 1 clinical trial of Temferon has started, and the company announced that drug-limiting toxicity was not observed at doses of 0.5, 1.0 and 2.0 × 10^6^ cells/kg and that higher dosages will be applied (https://ir.genenta.com/news-releases/news-release-details/genenta-progresses-higher-dosing-cohort-temferontm-phase-12a). MCY-M11 is a CAR-macrophage that expresses a mesothelin-targeting chimeric antigen receptor via flow electroporation of mRNA, and it was evaluated in a phase 1 clinical trial for multiple solid tumors (NCT03608618). The company announced that no dose-limiting toxicity or safety concerns were observed in the dose escalation cohort of 15 treated patients, and the feasibility of the manufacturing process was confirmed (https://investors.maxcyte.com/news-releases/news-release-details/phase-i-clinical-trial-mcy-m11-progressed). However, the following study was terminated because the sponsor shifted in focus (NCT03608618). A company, Carisma, developed CAR macrophages, which contain HER2-targeted chimeric antigen receptors and are designed for treating HER2-overexpressing solid tumors (https://www.nature.com/articles/d43747-020-01096-y). It suppressed tumor growth in a HER2+ ovarian cancer mouse model in vivo [407]. A phase 1 clinical trial using anti-HER2 CAR-Ms as monotherapy or in combination with pembrolizumab (NCT04660929) is ongoing.

Another strategy for the use of macrophages as a cancer therapy tool is the induction of cytokine secretion by macrophages. Hematopoietic stem/progenitor cells were isolated and transduced with a lentiviral vector encoding IL-12 in medium containing SCF, IL-6, and FLT3-L, which turned into IL12-GEMy (genetically engineered myeloid cells) [408]. IL12-GEMy reversed immune suppression by activating antigen-presenting cells and T cells after intravenous injection, which reduced metastasis and improved the survival of tumor-bearing mice. Recently, some researchers have established agents to program macrophages in situ. A nanoporter (NP)–hydrogel superstructure was constructed for locoregional in situ induction of CD133-specific CAR-MΦs in the tumor resection cavity, which minimized the systemic adverse effects of macrophage reprogramming. Nanoporter (NP)–hydrogel-engineered CD133-CAR-MΦs in situ promoted an antitumor immune response, which prolonged the survival of glioma-bearing mice. A lentiviral vector platform was established to deliver the IFNa coding sequence to liver macrophages [409]. The engineered liver macrophages expressed IFNa and activated T cells, which inhibited liver metastasis in mice. An oncology company called Myeloid Therapeutics developed in vivo engineering technology to reprogram myeloid cells expressing TROP2-CAR, which enables macrophages to recognize and kill tumor cells. A phase 1 clinical trial for epithelial tumors was initiated in 2023 (NCT05969041).

Elegant engineering of a family of chimeric antigen receptors for phagocytosis (CAR-Ps) has demonstrated high efficiency in the antigen-specific phagocytosis of both antigen-coated synthetic particles and antigen-expressing cancer cells [410]. The CAR-Ps were constructed from the CAR-P molecules containing the extracellular single chain antibody variable fragment (scFv) recognizing the B-cell antigen CD19 (aCD19) and the CD8 transmembrane domain present in the aCD19 CAR-T cells, and their phagocytic efficiency was enhanced by the cytoplasmic domains from Megf10 and FcRɣ. In the case of expression of unique antigens by cancer cells, such approaches can be very efficient. Overall, cell-based therapy in the case of genetically engineered TAMs can offer more localized effects, avoiding the frequent occurrence of systemic off-target complications of chemically or antibody-based anticancer drugs. In this context, epigenetic editing offers a new level of anticancer macrophage engineering.

## Concluding remarks

The knowledge we accumulated regarding the molecular mechanisms that are utilized by cancer cells to program TAMs to support tumor growth and spread yielded in the number of TAM-targeting approaches that have been validated or are under validation in clinical trials. Clinical trials have demonstrated that the survival, viability, differentiation and migration of monocytes and macrophages result in severe adverse or rebound effects, whereas monocytes and macrophages are the principal cells for the immunological integrity of the whole organism, even in the absence of infections. More promising approaches are those that try to reprogram specific TAM functions with tumors by blocking their protumoral or antitumor activity, where the induction of inflammatory or phagocytic activities is most favored. The greatest challenge in TAM-targeting therapies is avoiding off-target effects since healthy macrophages distributed in all tissues in the body can express the same molecules that are targeted by TAMs. A growing number of cancer-specific TAM biomarkers can help achieve the necessary level of precision in the delivery of drugs to the desired TAM subpopulation; however, double or even triple determinants on TAMs must be considered when drug delivery systems are designed. One more approach that has not yet been developed for TAMs can be epigenetic editing, where CRISPR‒Cas epigenetic enzymes can be delivered to specific promoters to edit DNA methylation or histone codes [411–413]. Recently, identified epigenetic programs of the protumoral functions of TAMs can be explored via such an epigenetic editing approach.
